# Revisiting the Anti-Cancer Toxicity of Clinically Approved Platinating Derivatives

**DOI:** 10.3390/ijms232315410

**Published:** 2022-12-06

**Authors:** Benjamin N. Forgie, Rewati Prakash, Carlos M. Telleria

**Affiliations:** 1 Experimental Pathology Unit, Department of Pathology, McGill University, Montreal, QC H3A 2B4, Canada; 2 Cancer Research Program, Research Institute, McGill University Health Centre, Montreal, QC H4A 3J1, Canada

**Keywords:** cisplatin, carboplatin, oxaliplatin, cellular uptake, DNA damage, transcription regulation, non-nuclear targets, chemoresistance, mechanisms of action, clinical usages, influx and efflux pumps, ribosome biogenesis, ER stress response, immunogenic cell death, interstrand and intrastrand DNA crosslinks

## Abstract

Cisplatin (CDDP), carboplatin (CP), and oxaliplatin (OXP) are three platinating agents clinically approved worldwide for use against a variety of cancers. They are canonically known as DNA damage inducers; however, that is only one of their mechanisms of cytotoxicity. CDDP mediates its effects through DNA damage-induced transcription inhibition and apoptotic signalling. In addition, CDDP targets the endoplasmic reticulum (ER) to induce ER stress, the mitochondria via mitochondrial DNA damage leading to ROS production, and the plasma membrane and cytoskeletal components. CP acts in a similar fashion to CDDP by inducing DNA damage, mitochondrial damage, and ER stress. Additionally, CP is also able to upregulate micro-RNA activity, enhancing intrinsic apoptosis. OXP, on the other hand, at first induces damage to all the same targets as CDDP and CP, yet it is also capable of inducing immunogenic cell death via ER stress and can decrease ribosome biogenesis through its nucleolar effects. In this comprehensive review, we provide detailed mechanisms of action for the three platinating agents, going beyond their nuclear effects to include their cytoplasmic impact within cancer cells. In addition, we cover their current clinical use and limitations, including side effects and mechanisms of resistance.

## 1. Introduction

During the first world war, the field of cancer treatment shifted from a surgical and radiology focus thanks to the use of the mustard gas [1]. A paper published in 1946 based on patients with non-Hodgkin’s Lymphoma going into remission after treatment with a mustard gas analogue pushed the research towards the development of chemotherapeutic compounds such as methotrexate, thiopurines, and 5-fluorouracil [2]. All previous drugs have in common that they are all organic chemicals; in the 1960s, however, the discovery of an inorganic, metallic compound with both cytostatic antibacterial and cytotoxic anticancer behaviour termed cisplatin exploded the field of anticancer research into a completely different avenue [3,4]. Although metal-based compounds were used since antiquity as treatment for many diseases, platinum-based compounds, such as cisplatin, showed cytotoxic benefit in cancer unlike any other metal, due to their higher selectivity, lower toxicity, and broader spectrum of activity [3,4]. Following cisplatin, several platinum (Pt)-analogues were developed, although only two were globally approved for usage in the clinic: carboplatin and oxaliplatin. A few others were approved only on a country-specific basis and will not be discussed here [5,6,7].

Since the approval of cisplatin in the late 1970s, Pt-based drugs have remained as prominent anticancer drugs, and are the dominant treatment for many solid tumours, including testicular, ovarian, bladder and colorectal cancers. Cisplatin, carboplatin and oxaliplatin are all considered to canonically act via the generation of DNA adducts which lead to subsequent DNA damage; however, the scope of their complete cytotoxicity within the cell is much broader and not limited only to the nucleus [6,7,8]. In this review, we summarize what is known about the three most popularly approved Pt-based drugs as follows: (1) usage of Pt drugs in clinical and research settings; (2) investigation of their mechanisms of action, including uptake, repair, and organelle-specific effects; (3) limitations of their utilization based on side effects and drug resistance.

## 2. Cisplatin: The First Platinating Agent

**Figure 1:**
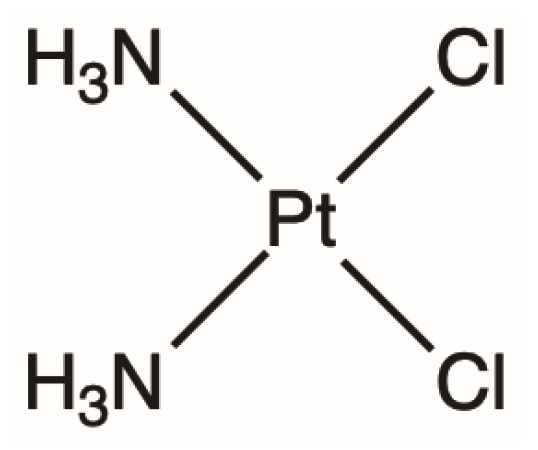
Structural formula of cisplatin. The molecule was adapted from the structure published in the CheBI database [13] using ChemDraw software (v21.0.0.28), PerkinElmer, Waltham, MA, USA.

Cisplatin, also referred to as cis-diamminedichloroplatinum (II) (CDDP) (Figure 1), is a widely used, Pt-based anti-cancer chemotherapeutic agent. This transition metal coordination complex has a square planar molecular geometry and presents as a solid, yellow powder at room temperature [9]. CDDP is fairly insoluble in most substances; however, it can be dissolved in dimethylformamide, and is somewhat soluble in water, but most preferable in saline sodium [9]. In solid form, CDDP is stable for about two years if stored at room temperature in dry conditions and light protected; however, it is known to convert slowly to its trans-form [9]. The stability of CDDP in sodium chloride solution is dependent on the chloride ion concentration; it is more stable when suspended in normal saline (0.9% sodium chloride) [10]. Saline also provides a reno-protective function, increasing hydration and aiding in excretion of CDDP from the kidneys [11,12].

Originally known as Peyrone’s salt, CDDP was first synthesized in 1844 by Michele Peyrone [14]. Despite its early discovery, the structure of CDDP was not determined until 1893 by Alfred Werner, and its potential as an anti-cancer agent was not elucidated until the 1960s by Barnett Rosenberg [15,16,17]. In 1965, Rosenberg sought to determine the effect of electric fields on the division of *Escherichia coli (E. coli),* but inadvertently rediscovered new properties of CDDP. Using Pt electrodes, Rosenberg and his team applied a current to a chamber containing a solution with *E. coli*; they found that the bacteria stopped dividing but continued to grow up to 300 times their normal size [15]. Over the following two years, Rosenberg determined that it was not the electric field causing this effect, but rather the electrolysis products formed from the Pt electrodes. A number of products were formed after the passing of a current; however, following chemical analysis, CDDP was found to be the active agent [15]. In 1969, Rosenberg expanded his work with CDDP into mammalian systems. Mouse models were used to determine the efficacy of CDDP against sarcoma-180 and leukemia L1210 tumours [16]. In both cases, intraperitoneal injection of CDDP caused a halt of cancer growth, decreasing mean tumour mass of the sarcomas, and increasing the mean survival time of the mice with leukemia [16]. The use of CDDP in clinical trials began in 1971 and yielded positive results in support of the compound as an anti-cancer drug [18,19]. While many researchers were in favour of using CDDP as a potential therapeutic agent, there were those that doubted its usefulness due to the toxicity associated with injection of the heavy metal. Neurotoxicity, nephrotoxicity, and ototoxicity were all among the list of concerns with utilizing CDDP for cancer treatment [20,21,22]. Despite the reservations regarding its toxicity, however, CDDP was first approved in 1978 by the United States Food and Drug Administration (US FDA) for its use against ovarian and testicular cancers [7]. Since its FDA approval, CDDP revolutionized cancer therapy, shifting the established cancer treatment paradigm towards heavy metal adjuvant therapy, and providing options for cancers once deemed untreatable [2].

### 2.1. Clinical Usage of Cisplatin

CDDP, despite being approved over 40 years ago, is still used today against many cancers. These include breast, ovarian, testicular, head and neck, esophageal, lung, bladder, and brain tumours [23] (Table 1).

The rise in the use of CDDP for treatment of these cancers resulted in a marked increase in survival, with testicular cancer as a particularly remarkable example. Prior to CDDP treatment, disease-free survival rates for testicular cancer ranged from 5 to 10%; however, with the introduction of CDDP in the 1980s, these rates drastically increased to 85% [24]. Today, testicular cancer is virtually curable in the early stages, with the disease-free survival hovering at 95% [25]. CDDP, in addition to working well as a cancer therapeutic on its own, also contributed to the practice of combination chemotherapy, becoming a base upon which, many mixed therapies were developed [9]. CDDP is currently approved in combination with over twenty other anti-cancer drugs for the treatment of a variety of cancers, and hundreds of trials are currently being performed using CDDP in combination with other drugs [9,26].

### 2.2. Cellular Uptake of Cisplatin

**Figure 2:**
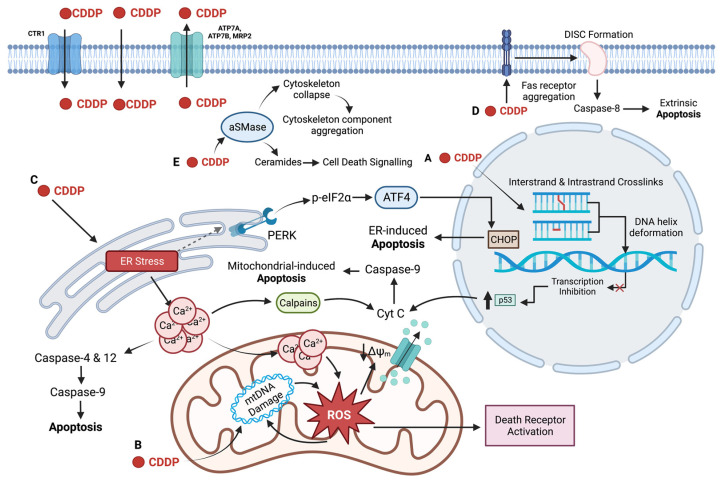
CDDP targets the nucleus, mitochondria, ER, plasma membrane, and cytoskeleton to induce cell death in its role as an anti-cancer drug. CDDP enters the cell via passive diffusion and high affinity copper uptake protein 1 (CTR1) and is removed from the cell by efflux transporters, ATP7A, ATP7B, and multidrug resistance protein 2 (MRP2). (**A**) CDDP forms intrastrand and interstrand crosslinks, distorting the DNA helix, inhibiting transcription (represented by the red cross) and activating p53-mediated apoptosis. (**B**) CDDP damages mitochondrial DNA (mtDNA), preventing transcription of electron transport chain (ETC) genes. Impaired ETC function results in ROS formation that further damages the mitochondria and activates the intrinsic and extrinsic apoptotic pathways. (**C**) CDDP induces the ER stress via the unfolded protein response (UPR), marked by upregulation of GRP78 and PERK-phosphorylation of eIF2α; ATF4 and CHOP are activated leading to ER-induced apoptosis. ER stress also leads to a release of calcium into the cytoplasm, which can directly activate caspase-12 and -4, as well as calpains, leading to apoptosis. High levels of calcium are also taken up by the mitochondria, depolarizing the membrane, resulting in apoptosis via cytochrome *C* release. (**D**) CDDP increases membrane fluidity and induces aggregation of components of the death inducing signalling complex (DISC) in the plasma membrane, resulting in extrinsic apoptotic activation. (**E**) CDDP activates acid sphingomyelinase (aSMase) which leads to cytoskeletal collapse and production of ceramides, signalling cell death. Created with BioRender.com (accessed on 11 November 2022).

CDDP is most commonly administered as cycles of IV injection given every three to four weeks. In the blood, roughly 90% of CDDP is sequestered by plasma proteins, leaving just 10% to access tissues [27]. The mechanism of CDDP uptake into cells is currently a subject of debate. Initially, it was thought that CDDP entered the cell solely through passive diffusion due to its relatively small size and neutral charge. Indeed, the ability of CDDP to diffuse through lipid bilayers has been shown extensively in vitro [28,29,30]. In addition, key pieces of evidence support the notion of a mainly passive mode of CDDP transport (Figure 2). First, it has been demonstrated that intracellular CDDP uptake remains unchanged upon co-treatment with analogs [31,32]. Human ovarian carcinoma cells were treated with CDDP along with excess carboplatin, transplatin, and cis-PdCl_2_(NH_3_)_2_. The presence of these structural analogs was unable to inhibit CDDP uptake, even when using concentrations 60-fold higher than that of CDDP in the case of carboplatin [31]. Cis-PdCl_2_(NH_3_)_2_ was the only compound capable of decreasing CDDP uptake, reducing accumulation by 20%. However, the authors suggest that this decrease was likely due to nonspecific damage from the reactive palladium compound, rather than to inhibition of a transport protein which would have had an expected decrease of 70% [31]. Additional support for passive diffusion comes from a 1973 study in which Gale et al. found that accumulation of cis-diammine(dipyridine) Pt (II) could not be saturated in Ehrlich ascites tumour cells. Indeed, the rate of uptake remained linear up until the compound reached its saturation limit in DMSO [28]. Since then, this finding has been confirmed by others using CDDP in other cell lines [33,34].

The idea that CDDP accumulation may involve more than simple passive diffusion, however, arose through studies of CDDP resistance. In 2002, Ishida et al. found a connection between the high affinity copper uptake protein 1 (CTR1) and CDDP resistance. CTR1 knockouts in yeast resulted in a decreased CDDP uptake and provided resistance to the cells, allowing them to grow in otherwise toxic doses of CDDP [35]. In addition, cells that were found to be resistant to copper transport were often found to be cross-resistant to CDDP, suggesting copper transporter involvement in CDDP uptake (Figure 2). Indeed, yeast with CTR1 knockouts were found to have a 30% decrease in CDDP adduct formation compared to wild-type cells following CDDP treatment [35]. Likewise, this phenomenon was mirrored in mammalian cell lines. Homozygous CTR1-knockout mouse ovarian cells were 8-fold more resistant to CDDP than wild-type cells, while heterozygous mutants displayed a 4-fold higher resistance [35]. Katano et al. similarly demonstrated cross-resistance to CDDP in copper transport resistant cell lines [36]. They found that CDDP-resistant cell lines had lower overall intracellular copper concentrations, and that these cells also showed lower rates of copper and CDDP accumulation [36]. Although these studies demonstrate a correlation between CTR1 activity and CDDP uptake, it is still debated whether increased CDDP uptake by CTR1 translates into increased toxicity. In fact, some argue that while CTR1 may transport CDDP, it does not significantly contribute to CDDP toxicity. Holzer et al., for example, found that increased CTR1 expression increased CDDP uptake in ovarian cancer cells, but that there was no resulting increase in cytotoxicity or DNA adduct formation [37]. Rather, they suggest that CTR1 mediated CDDP transport results in sequestration of CDDP in such a way that it cannot access the DNA, preventing CDDP transported by CTR1 from contributing to cytotoxicity [37]. Holzer et al. subsequently demonstrated that treatment with CDDP actually leads to degradation of both exogenous and endogenous CTR1 on the plasma membrane, a phenomenon that was also observed upon copper treatment [38,39]. However, the internalization and degradation of CTR1 following CDDP treatment have been disputed by other groups [33,40,41]. It is even argued that CTR1 does not act at all as a CDDP transporter. Kalayda et al. and Beretta et al. found that increased expression of CTR1 did not increase CDDP influx in ovarian carcinoma cells and cervical carcinoma cells, respectively, in direct opposition to previous work in support of CDDP uptake by CTR1 [40,42]. Another important point to consider is that CTR1 mediated transport of CDDP seems to be tissue specific [33,43]. For example, out of five small-cell lung cancer cell lines, only one showed a correlation between decreased CTR1 levels and increased resistance to CDDP [44].

Another transporter seemingly implicated in CDDP accumulation is a Na^+^/K^+^-ATPase. In 1991, Andrews et al. found that CDDP uptake in OV2008 human ovarian carcinoma cells was decreased by 50% upon treatment with the Na^+^/K^+^-ATPase inhibitor ouabain [45]. In addition, they found that depleting ATP reduced CDDP accumulation [45]. This result has since been replicated by other groups. In 2006, Kishimoto et al. compared a parental CDDP-sensitive H4-II-E cell line with a CDDP-resistant H4-II-E cell line. They found through Western blotting that the CDDP-resistant cell line expressed lower levels of the Na^+^/K^+^-ATPase alpha subunit [46]. In addition, upon depleting ATP using antimycin A, CDDP accumulation was decreased in the sensitive cells, but not in the resistant cells [46]. This evidence suggests a potential role for active transport in CDDP accumulation that may be lost as a mechanism of CDDP resistance [46]. Interestingly, Andrews et al. noticed in their study that the net Na^+^/K^+^-ATPase levels and activity were virtually identical in CDDP-sensitive and -resistant cells [45]. They also found that CDDP accumulation was partially sodium dependent, and that it may depend on the electrochemical gradient maintained by Na^+^/K^+^-ATPase, in addition to the active CDDP transport [45]. Incubation in low sodium media or other conditions that disrupted the sodium gradient resulted in a significant decrease in CDDP accumulation [45]. Likewise, excess potassium resulted in a drastic 5-fold increase in CDDP influx, suggesting a relationship between the membrane polarization state and CDDP accumulation, in which membrane depolarization increases CDDP uptake [47]. This relationship was further examined by Andrews et al. who showed that two CDDP-resistant cell lines had elevated basal membrane potentials in comparison to CDDP-sensitive cell lines [47]. In summary, CDDP transport is likely a combination of passive, facilitated, and active transport mechanisms. However, it is unknown how much of a role each mechanism plays in overall CDDP transport. Further work must be carried out to reconcile the conflicting evidence presented in this review, as CDDP transporters could present as an attractive target for CDDP-resistant cancer cases.

### 2.3. DNA as the Primary Target of Cisplatin

The most well-studied mechanism of CDDP-mediated cell death is nuclear DNA damage following intracellular uptake. In the plasma, CDDP is relatively unreactive as the high chloride concentration (~100 mM) prevents the displacement of the chloride leaving groups [48]. The intracellular chloride concentration, however, is much lower, varying between 5 and 60 mM. The decreased chloride concentration within the cell favours the aquation of CDDP, resulting in a di-aquo species [49,50,51]. The resulting compound, (Pt(H_2_O)_2_(NH_3_)_2_)^2+^, is a powerful electrophile that can bind a variety of cellular constituents, one of which being the highly nucleophilic N7-sites on the purine bases of the DNA [50]. CDDP can fit well into the major groove of the DNA helix, allowing easy access for adduct formation [50]. The major adducts formed by CDDP can be grouped into three major types: intrastrand crosslinks, interstrand crosslinks, and mono-functional adducts [50] (Figure 2A). The majority (~90%) of CDDP adducts are intrastrand crosslinks; 65% are 1,2-d(GpG) intrastrand crosslinks, 25% are 1,2-d(ApG) intrastrand crosslinks, and 5% are 1,3-d(GpXpG) intrastrand crosslinks. The remaining 5% of adducts are comprised of 2–5% interstrand d(GpG) crosslinks, and less than 1% are CDDP mono-functional adducts [50,52,53,54]. As the majority, intrastrand crosslinks are commonly thought to be the primary lesion responsible for CDDP cytotoxicity; however, the roles of interstrand crosslinks and mono-functional adducts have not been fully elucidated, and therefore, cannot be discounted when considering CDDP-mediated cell death [9]. Indeed, recent studies provide evidence for interstrand crosslinks as significant contributors to CDDP toxicity and will be discussed later.

The ability of CDDP to inflict DNA damage has been known since the 1970s and is considered the principal mechanism of CDDP-mediated toxicity [23,55,56,57,58]. Yet, the exact mechanisms of how CDDP DNA damage results in cell death remain unclear, in part due to the complex and multifaceted nature of how CDDP causes damage to the cell, and how the cell chooses to respond in turn. One long contested subject is the importance of replication and transcription stalling in CDDP-mediated cell death. Initially, it was thought that the main mechanism of cell death was inhibition of DNA synthesis, achieved through blocking DNA replication machinery by CDDP-DNA adducts [59,60]. In the late 1980s, studies by Sorenson and Eastman shifted this established paradigm away from inhibition of DNA synthesis towards prevention of RNA transcription as the primary culprit of CDDP related cell death [61,62,63]. The results of these studies made two important points that casted doubt on the inhibition of DNA synthesis as a mechanism of CDDP-mediated cell death. First, cells treated with CDDP typically arrest within the G2 phase of the cell cycle, not the S phase as would be expected with inhibition of DNA synthesis [61]. Second, in studies of DNA repair-deficient mouse ovarian cell lines, treatment with doses of CDDP known not to inhibit DNA synthesis were still found to be capable of inducing G2 growth arrest and apoptosis [62]. In addition, they found that DNA was able to double even in cells that did not divide [61,63]. Rather, Sorenson and Eastman point to inhibition of transcription as the key mechanism of CDDP toxicity, suggesting that G2 arrest is a result of decreased whole-cell RNA and protein levels [61,63].

### 2.4. Regulation of Transcription by Cisplatin

The ability of CDDP to inhibit transcription by RNA polymerase II (Pol II) has been widely documented [64,65,66,67,68,69,70]. Corda et al. provided early evidence of this phenomenon in the 1990s. They showed that, in vitro, transcription of a template strand was blocked by CDDP adducts, while the complementary strand was able to be transcribed [67]. Later, they determined that ribonucleotide addition was differentially affected by different CDDP-DNA adducts. For example, d(GpG) intrastrand crosslinks inhibited Pol II to a higher degree than d(ApG) crosslinks [68]. It was determined that d(GpG) lowered the binding affinity of Pol II to the DNA template, while no such effect was observed with d(ApG) crosslinks [68]. Comparing interstrand and intrastrand crosslinks revealed that both types of CDDP adducts where capable of diminishing single nucleotide addition by pol II, and that both were able to irreversibly blocking nucleotide elongation [69]. Interestingly, both the cis- and trans- isomers of DDP can inhibit DNA replication, decreasing DNA synthesis to a similar degree. Yet, the trans isomer is significantly less potent and dangerous to cancer cells than CDDP [70]. This provides further evidence that inhibition of DNA replication is not the main culprit in CDDP-mediated cell death. Indeed, in 1995, Mello et al. provided in vivo evidence that CDDP blocked transcript elongation of a plasmid template in mammalian cells 2-fold more than transplatin [70]. Furthermore, they provided evidence that the difference between CDDP and transplatin was not due to preferential repair of transplatin lesions by nucleotide excision repair (NER) or due to a decreased number of transplatin adducts compared to CDDP [70]. Finally, 60–76% of transplatin adducts could be bypassed by Pol II, while only 0–17% of CDDP adducts could be bypassed by the enzyme [70].

**Figure 3:**
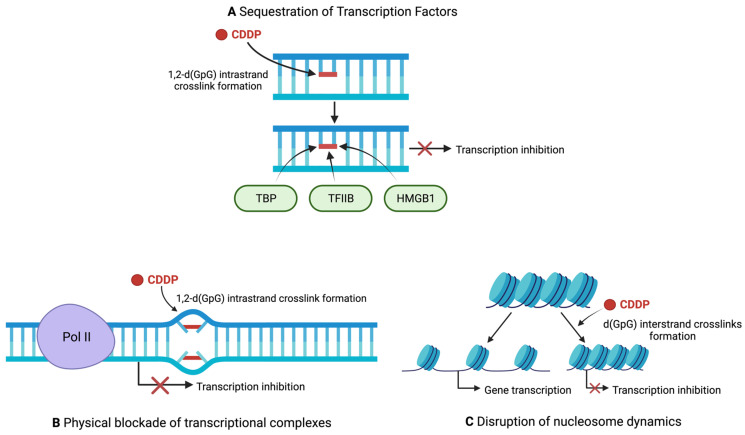
Mechanisms of transcription inhibition by CDDP. (**A**) CDDP hinders transcription by sequestering transcription factors away from promoter regions. (**B**) Bulky CDDP lesions act as a physical roadblock to transcription machinery, preventing transcript elongation. (**C**) CDDP interstrand crosslinks hinders nucleosome mobility, preventing chromatin remodelling and maintaining condensed chromatin that prevents transcription. Red crosses represent inhibition of transcription. Created with BioRender.com (Accessed on 11 November 2022).

CDDP damage decreases transcription levels through three mechanisms: sequestration of transcription factors, physical blockade of transcriptional complexes, and through chromatin alterations [71] (Figure 3). In 1997, Vichi et al. were able to prove, through in vitro experiments utilizing reconstituted transcriptional systems, that the presence of CDDP-damaged DNA resulted in a 4-fold reduction in transcription compared to an undamaged template [72]. Indeed, this finding was again validated by another group in 1999, providing further evidence that transcription can be repressed by CDDP-DNA adducts [73]. Moreover, the TATA binding protein (TBP) was shown to directly bind CDDP-DNA adducts in nitrocellulose filter binding assays, and incubating CDDP-damaged DNA with TBP in a recombinant transcriptional system drastically reduced transcription of the undamaged template [72]. Even when the template DNA contained TATA box motifs, TBP still preferentially bound CDDP-DNA adducts and only by re-introducing excess TBP into the assay reaction was the transcriptional repression alleviated [72]. This suggests that CDDP-damaged DNA acts as a competitive inhibitor of the TBP–TATA box interaction and interferes with transcription through sequestering essential transcriptional machinery [72] (Figure 3A). This phenomenon was also seen with other components involved in the transcription complex such as TFIIB and TFIIH, however to a somewhat lesser degree than with TBP [72]. Sequestration by CDDP-damaged DNA has since been demonstrated with other transcription factors. For example, the rRNA transcription factor known as the upstream binding factor (UBF) is also sequestered by CDDP-DNA damage [74,75]. UBF tightly binds 1,2–dGpG intrastrand crosslinks, preventing rRNA transcription in vitro [74,75]. In this way, sequestering UBF could halt the production of rRNA, a necessary process during cell proliferation and growth, and contribute to CDDP toxicity [75]. In addition, the Y-box, high mobility group box 1 (HMGB1), and the structure specific recognition protein 1 (SSRP1) in complex with Spt16 are all important transcription factors that have each been shown to specifically bind to CDDP-DNA adducts with high specificity, and likely contribute to transcription inhibition by CDDP [76,77,78] (Figure 3A).

Initial evidence for the physical blocking of transcription machinery directly by CDDP adducts was provided by Mymyrk et al. in 1995, when they showed that CDDP adducts could block NF1 binding to template DNA [79]. Indeed, in vitro studies confirmed that CDDP-damaged DNA could block NF1 transcription factor binding. The template DNA utilized had no chromatin structure, confirming that CDDP was not inducing a change in chromatin conformation that blocked NF1 binding in this experiment [79]. In other words, CDDP-adducts alone were shown to be sufficient to directly block transcription factor binding, either through steric hindrance or through DNA helix distortion [79]. A clearer picture of how CDDP blocks Pol II came from a structural study of yeast Pol II in complex with a CDDP-damaged nucleotide scaffold [80]. In this study, a d(GpG) intrastrand lesion was specifically placed on the +2/+3 position of the template scaffold, with the +1 position being where ribonucleotide addition takes place. The authors found that the bulky CDDP lesion prevent the afflicted base from being stably accommodated in the Pol II active site. Indeed, backtracking of the polymerase occurred when the lesion was attempted to be placed in the active site [80]. The authors therefore proposed that CDDP acts as a translocation barrier of sorts, preventing the adducted base from correctly binding the active site of Pol II, thereby physically stopping Pol II in its tracks [80] (Figure 3B). Another observation was the misincorporation of AMP by the Pol II elongation complex at the site of lesion, creating a G-A mismatch [80]. The phenomenon of AMP misincorporation by stalled polymerases is well documented, and is known as the A-rule [81]. However, Pol II stalling was shown to occur regardless of the presence of the G-A mismatch, further suggesting a bulky lesion translocation barrier as the main feature in Pol II stalling [80].

The final mechanism thought to contribute to transcriptional inhibition is the disruption of nucleosome dynamics. The condensation of eukaryotic DNA into chromatin provides a dual function, allowing the DNA to fit within the nucleus of the cell acting as a level of regulation for gene expression [82]. The nucleosome is the base unit of chromatin structure and consists of DNA coiled around histone core proteins. The wrapped DNA becomes inaccessible to the transcription machinery, and thus, chromatin remodelling through nucleosome mobility is critical for ensuring successful gene transcription [83]. CDDP has been shown to interfere with nucleosome dynamics and chromatin remodelling both in vitro and in vivo (Figure 3C). As early as 1995, observations were made that CDDP treatment in mouse mammary cells prevented both nucleosome shifting and transcription itself; however, a mechanistic explanation of how CDDP prevented nucleosome mobility was not given [79]. More recently, structural studies have been able to provide some insight into the mechanism of how CDDP locks nucleosomes. X-ray crystallographic studies by Ober and Lippard have shown that intrastrand crosslinks positioned inside the nucleosome adopt a position facing inwards, toward to the core [84,85]. They suggest that the sliding of nucleosomes along Pt-damaged DNA would bend the lesion into an unfavourable position [86]. In this way, CDDP crosslinks can lock nucleosomes in place and prevent chromatin remodelling. Interestingly, 1,2 intrastrand crosslinks were found to have a stronger inhibitory effect on nucleosome mobility than 1,3 crosslinks, as the 1,2 crosslinks bend at a more severe angle in the nucleosome [86]. Yet, despite being able to reduce nucleosome mobility, adducting nucleosome DNA with CDDP was unable to prevent transcription by bacterial RNA polymerases. This finding led Ober and Lippard to conclude that nucleosome dynamics must not be a mechanism of transcriptional inhibition by CDDP [86]. Others, however, would disagree with this conclusion. In a 2021 study, Moon and colleagues found that CDDP locks nucleosomes in place and fastens chromatin. Even in conditions of high salt and mechanical force, which can release untreated nucleosomes, CDDP treatment resulted in nucleosome fixation, fully preventing the disassembly of the histones [87]. This fixation also seemed to be permanent, remaining even after exposure to 3M NaCl [87]. Important to note, however, is the concentration of CDDP used in this study. Treatments with 3.3 mM CDDP, and to a lesser extent 0.1 mM, were capable of locking nucleosomes and suppressing transcription of genes contained within the nucleosomes [87]. The therapeutic range for CDDP is thought to lie between 1.0 and 5.0 mg/mL, or roughly between 3 and 16 µM [88]. Concentrations of 3.3 mM and even 0.1 mM are therefore much too high to be physiologically relevant and must be considered when analyzing these findings. Another question to consider is how intrastrand and interstrand crosslinks differ in their ability to suppress nucleosome mobility. In a 2013 study by Zhu, Song and Lippard, nucleosome shifting was observed upon heat treatment. Nucleosomes pretreated with CDDP, however, had a drastically reduced ability to heat shift [89]. Interestingly, nucleosomes with 1,2-d(GpG) intrastrand crosslinks were readily shifted upon heat exposure, while nucleosomes with interstrand crosslinks demonstrated a significantly reduced ability to shift [89]. However, both types of crosslinks were capable of inhibiting transcription in vivo regardless of their ability to prevent nucleosome shifting [89]. These results point to a potential mechanistic difference between CDDP intrastrand and interstrand crosslinks. Perhaps reduced nucleosome mobility is a mechanism used by interstrand, but not intrastrand, crosslinks to inhibit transcription. Alternatively, reduced nucleosome mobility may not play a significant role in the inhibition of transcription, acting because of CDDP-mediated damage rather than a cause. In either case, these results provide evidence that interstrand crosslinks may play a larger role in CDDP-mediated toxicity, a role that historically was attributed solely to intrastrand crosslinks.

How CDDP mediates cell death depends not only on the DNA lesions themselves but also on how the cell chooses to respond to the damage. In cases of short-spanned tolerable damage, the cell can activate cell cycle arrest and repair mechanisms. In cases of unmitigated sustained damage, the cell can activate pro-apoptotic pathways [90]. DNA damage by CDDP is recognized by various sensor proteins, including but not limited to HMGB1, hMutSa, hUPB, and TBP [91,92,93]. Transcriptional inhibition itself may also act as a pro-apoptotic damage signal, as it may increase the ratio of pro-apoptotic transcripts—which tend to have longer half-lives—to anti-apoptotic transcripts [71]. The recognition of CDDP DNA damage by these sensors leads to the activation of both intrinsic and extrinsic apoptotic pathways. In brief, CDDP damage results in action of the ATR kinase, which phosphorylates serine-15 on p53, resulting in its activation [94]. P53 can also be activated via ERK, which itself is activated by MAPK upon CDDP damage. Ultimately, activation of p53 results in upregulation and activation of pro-apoptotic proteins, which result in cytochrome *C* release from the mitochondria and activation of caspase-9 [95]. Caspase-9 will in turn activate the executioner caspases-3 and -7 leading to cell death [96,97]. CDDP may also activate a p53-independent form of intrinsic apoptosis that stems from the activation of the c-abl tyrosine kinase, resulting in the activation of p73 [98]. P73, a p53-like protein, activates intrinsic apoptosis similarly to p53, through cytochrome *C* release and activation of executioner caspases [98]. Finally, CDDP induction of MAPK also triggers extrinsic apoptosis through the JNK and p38 pathways, which culminate in Fas ligand (FasL) gene expression [99,100]. FasL in turn binds the Fas receptor (FasR) on the plasma membrane, resulting in the formation of the death-inducing signalling complex (DISC) followed by apoptosis [99,100].

### 2.5. Non-Nuclear Targets of Cisplatin

While DNA damage is historically considered to be the primary mechanism of CDDP-induced cell death, there is evidence to suggest that CDDP may induce apoptosis independently of nuclear DNA (nDNA) damage. For instance, enucleated HNSCC cell cytoplasts remain as sensitive to CDDP as their nucleated parental cell line [101]. In addition, it has been reported that CDDP can lead to caspase-3 activation even in enucleated cytoplasts, suggesting possible apoptotic initiation in the cytoplasm rather than in the nucleus [102,103]. In fact, in testicular germ cell tumours the DNA damage response proteins ATM, ATR, and DNA-PK are not required for initiation of apoptosis in response to CDDP treatment [104]. Thus, it is necessary when evaluating CDDP-mediated cell death to look for other cellular targets. One target that plays a large role in CDDP toxicity is mitochondrial function (Figure 2B). Interestingly, CDDP has a higher propensity for forming mitochondrial DNA (mtDNA) adducts than nDNA adducts, with mtDNA lesions being present in levels 300–500 fold higher than nDNA lesions [101]. Furthermore, in Chinese hamster ovary cells, there is not only higher initial binding of CDDP to mtDNA than nDNA, but also a lower rate of excision of mtDNA adducts than nDNA adducts, resulting in higher levels of mtDNA damage compared to nDNA [105]. Likewise, cells that have been depleted of their mtDNA are 4–5-fold more resistant to CDDP than their parental cell lines [101,106].

Mitochondrial damage via CDDP adducts on mtDNA results in oxidative stress and the production of reactive oxygen species (ROS) [107,108,109,110]. The production of ROS by CDDP has shown to be majorly dependent on mitochondrial damage (Figure 2B). Cells with depleted mtDNA do not generate nearly as high levels of ROS upon exposure to CDDP, and ROS generated following CDDP treatment is localized to the mitochondria [110,111]. In addition, pre-treatment of cancer cell lines with N-acetyl cysteine (NAC), an antioxidant, reduces sensitivity to CDDP alleviating its cytotoxic effects. CDDP adducts on mtDNA block mtDNA transcription, leading to a reduction in the translation of proteins necessary for the electron transport chain (ETC) [112]. Impaired ETC function can in turn generate high levels of mitochondrial ROS, which can damage a variety of cellular constituents including mtDNA, proteins, and lipids. ROS can further impair the mitochondria and the ETC, causing a positive feedback loop generating even more ROS and culminating in apoptosis via the intrinsic or extrinsic pathways [110,113,114]. For example, ROS can increase mitochondrial membrane permeability, disrupt mitochondrial membrane potential, and facilitate cytochrome *C* release from the mitochondria through activation of Bak and Bax [113,114,115,116,117]. ROS can also trigger the extrinsic apoptotic pathway by activating the death receptors TRAIL-R1/2, FasR, and TNF-R1 on the plasma membrane [113].

A 2019 study by Kleih and colleagues gave further insight into the mechanisms of CDDP-mediated mitochondrial ROS generation. They determined, in ovarian cancer cell lines, that CDDP-sensitive cells tended to have higher baseline mitochondrial content and mitochondrial ROS than CDDP-resistant cells [118]. Moreover, they found that treatment with CDDP actually stimulates mitochondrial biogenesis and suggest that CDDP treatment triggers an increase in mitochondrial content and subsequent increase in mitochondrial ROS production [118]. Indeed, preventing mitochondrial biogenesis through knockdown of PGC1a or TFAM, important factors for mitochondrial biogenesis, resulted in reduced susceptibility to CDDP [118]. This is paralleled by the fact that higher mitochondrial content, represented by high TFAM expression, correlates with a better 5-year survival in ovarian cancer patients [118]. However, this study was conducted only in ovarian cancer and should be repeated in other cancer types. In addition, it is unclear whether ROS production from CDDP treatment is a result of solely increased mitochondrial content, through damaged ETC, or a combination of both [118].

The endoplasmic reticulum (ER), an arm of the secretory pathway involved in protein folding and quality control, lipid synthesis, and calcium storage/homeostasis represents a cytoplasmic target of CDDP [119,120] (Figure 2C). As part of its role in maintaining protein quality control, the ER is equipped with mechanisms to overcome disturbances to protein homeostasis. One such mechanism is the unfolded protein response pathway that becomes activated in response to ER stress [121]. Under normal conditions, the ER chaperone GRP78 acts to facilitate proper protein folding, as well as negatively regulate UPR stress sensors [122]. However, upon ER stress, GRP78 becomes activated, in turn activating transcription factor 6 (ATF6), inositol-requiring protein 1α (IRE1α), and protein kinase RNA-like endoplasmic reticulum kinase (PERK) [122]. These stress sensors initiate responses that promote maintenance of protein homeostasis and cell survival, or in cases of extreme unmitigated ER stress, apoptosis [121,123]. For example, PERK acts to reduce global protein translation by phosphorylating eukaryotic translation factor 2α (p-eIF2α), while IRE1α initiates selective degradation of certain mRNAs. Both of these sensors work to reduce the total amount of protein moving through the ER, allowing the ER to remediate the imbalance of unfolded proteins and promote cell survival [123,124]. In cases of chronic ER stress, ATF4, a downstream factor of the PERK pathway, upregulates the expression of C/EBP homologous protein (CHOP). CHOP promotes apoptosis through inducing expression of pro-apoptotic factors while simultaneously downregulating anti-apoptotic factors [124,125]. In addition, IRE1α activation results in the activation of caspase-12, an ER-specific caspase that can induce apoptosis upon prolonged ER stress via activation of caspases-9 and -3 [126,127]. ATF6, upon ER stress, translocates to the Golgi apparatus, where it is subsequently cleaved and transported to the nucleus. In the nucleus, the cleaved portion of ATF6 induces expression of different UPR effectors, including ER chaperones and CHOP [128]. CDDP has been shown to induce aggregation of misfolded proteins within the ER, pushing the cell towards ER stress [129] (Figure 2C). Likewise, CDDP upregulates the expression of UPR markers, such as GRP78, caspase-12, GADD34, and CHOP [102,129,130]. Interestingly, this effect has been observed both in various cancer cells lines and in enucleated cytoplasts, confirming again that CDDP can trigger the cytoplasmic apoptotic pathway independent of nuclear damage [102]. Similarly, inhibition of caspase-12 activity has been found to reduce the percentage of apoptotic cells upon treatment with CDDP compared to controls, further implicating ER stress and caspase-12 activity as a notable mechanism of CDDP-mediated cell death [131].

ER stress induced by CDDP also contributes to cytotoxicity through the efflux of stored calcium from the ER into the cytoplasm, drastically increasing its cytoplasmic concentration [132]. Increased calcium in the cytoplasm leads to activation of calpains, calcium dependent cysteine proteases, as well as caspase-4 [133] (Figure 2C). Calpains aid in triggering cytochrome *C* release through the activation of the pro-apoptotic factor Bid, and are also thought to play a role in caspase-4 activation, both of which ultimately result in apoptosis [134]. Indeed, blocking calcium release from the ER through inositol triphosphate receptor inhibitors proved to prevent caspase-4 and calpain activation, while simultaneously mitigating apoptotic activation by CDDP [132]. Furthermore, increased calcium in the cytoplasm can cause calcium uptake into the mitochondria through various transporters [135]. Inside the mitochondria, the increased calcium concentration leads to the generation of ROS, which further damages the mitochondria and activate apoptosis [135,136].

Despite previous evidence, much remains to be understood regarding the role of ER stress in CDDP cytotoxicity, as certain studies have shown that ER stress can play a protective role against CDDP, rather than a cytotoxic one [120,130,137]. In a 2017 study, the HIV protease inhibitor saquinavir, known to induce ER stress in ovarian cancer cells, increased the IC_50_ of CDDP when used in combination [138]. Because of inducing ER stress, saquinavir also upregulated the expression of genes involved in autophagy, a common response to ER stress [138,139]. The authors suggest that increased autophagy resulting from ER stress assists in protecting the cell from CDDP by aiding in clearance of damaged cellular constituents [138]. Taken together, it is likely that ER stress affects the action of CDDP depending on the degree of ER stress. In low levels, ER stress seems to play a protective role, ameliorating the toxic effects of CDDP by inducing autophagy and other pro-survival pathways. In cases of consistent, high-level ER stress, however, cell death mechanisms in response to CDDP are the favoured response. Further investigation should be done on the interplay between ER stress and CDDP activity, as the ER stress response could provide a promising target for cancers with low sensitivity to CDDP.

Other cytoplasmic targets of CDDP are cellular lipids, particularly those of the plasma membrane. CDDP has been shown to interact with phospholipids and phosphatidylserine both in liposome membrane models and in human erythrocytes [140,141]. In fact, human erythrocytes change shape as a response to treatment with CDDP, indicating that CDDP may affect plasma membrane structure [142]. Yet, despite the interaction of CDDP with the erythrocyte membrane, and subsequent change in membrane integrity, there was no change in the fluidity of the membrane [142]. However, other studies have demonstrated changes in membrane fluidity because of CDDP treatment. Studies utilizing liposomes reveal, via atomic force spectroscopy, that CDDP induced a reduction in membrane fluidity compared to untreated liposomes [143]. In addition, lateral diffusion within the lipid bilayer has also been shown to decrease upon treatment with CDDP [144]. In contrast, studies involving HT29 human colon cancer cells provide evidence that CDDP actually leads to an increase in membrane fluidity [145,146]. Indeed, treatment with CDDP resulted in a rapid increase in membrane fluidity due to CDDP-mediated activation of acid sphingomyelinase (aSMase) activity [145,146]. Additionally, CDDP induced the formation and activation of the death-inducing signalling complex (DISC) on the plasma membrane. CDDP has been shown to cause aggregation of the Fas death receptor (FasR), a component of the DISC, in the plasma membrane [145]. Furthermore, CDDP causes accumulation and re-localization of pro-caspase-8 and FADD, other DISC components, along with the FasR, to lipid rafts. In this way, CDDP is likely inducing ligand independent activation of DISC, resulting in capase-8 activation and apoptotic signalling [145] (Figure 2D). Indeed, treatment with nystatin, a compound that sequesters cholesterol, prevented the redistribution of FasR, FADD, and pro-caspase-8 to lipid rafts upon CDDP treatment, and reduced CDDP-mediated cell death as a result [145]. The increase in membrane fluidity resulting from CDDP treatment is thought to aid in the redistribution of DISC components into the lipid rafts [145]. In fact, pre-treatment with cholesterol, which decreases membrane fluidity, resulted in the reduced re-localization of DISC components to lipid rafts and reduced apoptosis even in the presence of CDDP, further demonstrating the importance of membrane fluidity in CDDP-mediated DISC activation [145]. Interestingly, analysis of the plasma membrane composition of CDDP-resistant and CDDP-sensitive cell lines reveal that the CDDP-resistant cells had higher levels of phosphatidyl choline and lower levels of cholesterol in their plasma membranes, decreasing the fluidity [147]. The decrease in membrane fluidity in CDDP-resistant cells may act as a compensatory resistance mechanism to protect against the increase in membrane fluidity caused by CDDP [147]. Finally, CDDP induction of aSMase activity results not only in increased membrane fluidity but also in the production of ceramides [145,146] (Figure 2E). Ceramides are cell death signalling lipids shown to be involved with Bax accumulation in the mitochondria and permeabilization of the outer mitochondrial membrane and activation of caspase-3, ultimately resulting in apoptosis [148]. In this way, production of ceramides by CDDP-mediated activation of aSMase may contribute to cytotoxicity by CDDP.

In addition to the plasma membrane, CDDP also induces damage to the cytoskeleton. Cancer metastasis demands efficient remodelling of the cytoskeletal components, microfilaments, intermediate filaments, and microtubules, in order to allow for cell migration and invasion [149]. CDDP has been shown to directly interfere with the cytoskeleton network, causing its collapse and subsequent aggregation in the cytoplasm [150] (Figure 2E). For example, CDDP causes vast remodelling of the actin microfilament network, causing loss of filopodia and an increase in cortical stress fibers [151]. In addition, CDDP causes the destabilization of actin filaments anchored to the plasma membrane, causing its re-localization to the cytoplasm. This effect is mediated through ezrin, an actin binding protein with roles in anchoring actin to the plasma membrane, that becomes deactivated by CDDP via CDDP-induced aSMase activity [151]. Indeed, knockdown of aSMase activity abrogates the actin and ezrin re-localization induced by CDDP. In addition, treatment with ceramides alone cause the same effects of ezrin inactivation as by CDDP, showing that ceramides produced by CDDP-activated aSMase are responsible for the disruption of the actin network [151].

CDDP also displays activity against microtubules. Through direct binding to tubulin, CDDP promotes tubule depolymerization [152]. Furthermore, CDDP prevents assembly of tubulin into microtubules, which causes accumulation of tubulin aggregates unable to participate in cellular functions such as migration and mitosis [153]. In fact, a study investigating the role of CDDP in cytoskeletal function showed that CDDP treatment increased cell stiffness, and decreased cell migration, invasion, and colony formation as a result of its cytoskeletal effects [154]. In this way, CDDP exerts anti-tumour activity by disrupting cytoskeletal remodelling.

### 2.6. Limitations of the Use of Cisplatin

The introduction of CDDP into treatment regimens revolutionized cancer therapy. However, CDDP has significant drawbacks that limit its usefulness as an anti-cancer agent. The most significant limitations of CDDP are its severe side effects, which manifest primarily through nephrotoxicity, ototoxicity, and neurotoxicity (Table 1). Among them, nephrotoxicity is considered the most significant dose-limiting factor for discontinuance of CDDP with roughly one-third of all patients developing acute kidney injury in response to CDDP treatment [155,156,157,158]. It has been shown that the kidneys accumulate higher levels of CDDP than any other tissues, and that CDDP action on the kidneys results in reduced glomerular filtration rate and increased serum creatinine levels, indicating impaired renal function [158,159]. Through the same mechanisms described above, CDDP triggers an apoptotic response in the tubular cells of the proximal and distal tubules of the nephron [160,161]. In addition, CDDP treatment results in ROS production that further contributes to the induction of apoptosis via activation of mitochondrial damage and the extrinsic apoptotic pathway [162]. Furthermore, CDDP has been shown to induce an acute inflammatory response in renal tubular cells. CDDP treatment releases pro-inflammatory mediators, with TNFα considered the major factor involved in CDDP-induced inflammation [163,164]. Additionally, TNFα initiates pro-apoptotic signalling through its interaction with TNFR2 [164,165]. Finally, CDDP significantly damages the renal vasculature. Through ischemic injury and induced vasoconstriction, CDDP decreases the glomerular filtration rate and brings about hypoxic injury [166,167].

Another dose-limiting side effect of CDDP is the resulting ototoxicity [168]. Anywhere from 20% to 70% of patients experience CDDP-related ototoxicity, with this effect being more prominent in children [168,169]. CDDP induces ototoxicity through induction of ROS and apoptotic cell death within the hair cells of the ear [170,171]. As a result, 40–80% of patients develop permanent hearing loss [172]. Interestingly, the OCT2 transporter has been reported to be involved in mediating both CDDP nephrotoxicity and ototoxicity, by transporting CDDP into both renal tubule cells and hair cells of the inner ear [173,174]. In fact, blocking OCT2 activity results in markedly reduced cellular uptake of CDDP by these cells in vitro, and reduced nephro- and ototoxicity in vivo [173,174]. OCT2, while expressed in these cells, is not typically expressed to a significant degree in most tumour types that CDDP is used to treat [173]. Therefore, OCT2 may prove to be a useful target for reducing off target toxicity without diminishing CDDPs cytotoxic effects against tumour cells.

Neurotoxicity is an additional side effect of CDDP with severe consequences for patients. CDDP has been shown to accumulate and cause damage to the dorsal root ganglia causing morphological changes and resulting in peripheral neuropathy [175]. Sensory neurons are more significantly affected by CDDP, with common symptoms being paresthesia and dysesthesia in the extremities [175,176]. Neuropathic symptoms can continue to worsen up to 6 months post-completion of CDDP treatment and recovery tends to occur very slowly, with some patients reporting neuropathy symptoms up to 20 years post-treatment [177,178,179].

Finally, hepatotoxicity and cardiotoxicity have also been observed upon CDDP treatment; however, these are considered rare [180,181] (Table 1). More general side effects include nausea and vomiting, diarrhea, fever, weight loss, and transient hair loss, among others [182,183].

Another major limitation of CDDP is acquired resistance following repeated exposure, which is facilitated by a variety of mechanisms [184]. One way in which cells become resistant to CDDP is through decreased CDDP accumulation [184]. Many studies have shown that CTR1, the copper transport protein involved in CDDP import, plays an important role in CDDP resistance. CDDP-resistant cell lines have shown to have decreased expression of CTR1, which results in decreased intracellular accumulation of CDDP [40,185]. CTR2 may also play a role in CDDP resistance by stimulating the cleavage of CTR1 by cathepsin L/B and further reducing accumulation of CDDP [186].

Increased CDDP efflux is another mechanism of acquired resistance [184]. CDDP efflux is mediated by the copper efflux transport proteins ATP7A and ATP7B (Figure 2). Elevated levels of ATP7A/B correlate with increased resistance to CDDP in vitro and with poor prognosis in patients [187,188,189]. In addition, knocking out ATP7A increases the efficacy of CDDP in vitro [190,191]. Finally, multidrug resistance-associated proteins, such as MRP2, also contribute to enhanced CDDP efflux (Figure 2). MRP2 works to export CDDP bound to the cellular antioxidant glutathione (GSH), and increased MRP2 expression contributes to CDDP resistance [192,193]. Similarly, increased drug detoxification through GSH activity also contributes to CDDP resistance [194]. GSH binds activated CDDP in order to deactivate and facilitate its removal via MRP exporters and other detoxifying enzymes [194]. GSH also provides resistance to CDDP independently of detoxification through its activity as a ROS scavenger [195]. By reducing the high levels of ROS induced by CDDP, GSH ameliorates the cytotoxic oxidative stress [195,196]. Cells resistant to CDDP have shown to have increased expression of GSH and additional enzymes involved in CDDP detoxification and ROS scavenging [197]. In fact, reducing intracellular GSH re-sensitizes resistant cells to CDDP [198]. In addition, increased levels of NF-E2-related factor 2 (Nrf2), a transcription factor involved in the regulation of GSH and MRP, are associated with increased CDDP resistance in vitro and in vivo [199,200,201]. Nrf2 also contributes to CDDP resistance through its interaction with the ubiquitin binding protein p62 [202]. P62 activation by Nrf2 upregulates autophagy and clearing of misfolded proteins via the proteasome, reducing ER stress and further increasing resistance to CDDP [203,204].

Increased DNA damage repair is another mechanism of CDDP resistance [205]. Intrastrand crosslinks are primarily repaired through the NER pathway [206,207]. Crosslink formation distorts the shape of the DNA helix, allowing them to be detected by a complex of xeroderma pigmentosum C (XPC) and RAD23B [208,209,210]. Upon detection of the lesion, XPC-RAD23B recruits transcription factor II H (TFIIH), a complex of multifunctional protein subunits [211]. The XPB subunit unwinds the helix, while XPD locates the lesion and, upon stalling at the site of the crosslink, signals for the recruitment of the pre-incision complex, consisting of RPA, XPA, and XPG [212]. ERCC1-XPF, a 5′-endonuclease, is then recruited to the protein scaffold by interaction with XPA, where it cuts the DNA strand 5′ to the lesion [213]. A 3′ incision is then made by XPG, and together this process is known as dual excision [214]. Following excision, the lesion is released with TFIIH and repair is completed by a series of DNA polymerases (d, k, or e.) [215,216]. Finally, the nick in the helix is repaired most commonly by DNA ligase I [206]. It has been shown that increased expression of NER-related genes is associated with increased CDDP resistance. This effect has been observed with a multitude of NER genes, such as XPF, XPA, and XPG; however, the strongest correlation is seen with ERCC1 [217].

Homologous recombination (HR) is another DNA repair pathway involved in CDDP lesion repair. The pathway for repair of CDDP interstrand crosslinks involves the formation of double strand breaks (DSB) in the DNA [205]. These DSBs are then in turn repaired by specialized DSB-repair pathways such as HR [205]. BRCA-1 and -2 are essential to HR function and promote DNA end resection and recruitment of other HR components [218]. Defects in these genes are relatively common in certain cancer types, and increase the sensitivity of tumour cells to CDDP [219]. However, it has been shown that, in ovarian cancer cells, HR can be rescued by subsequent mutations that restore BRCA1/2 wild type activity, increasing resistance to CDDP as a result [220,221].

Finally, the mismatch repair (MMR) pathway, a DNA repair response that corrects single nucleotide mismatches, can also recognize CDDP lesions [222,223]. Interestingly, MMR cannot effectively repair CDDP adducts, as it causes replacement of the base opposite to the lesion rather than the damaged based itself [224]. As a result, the original damage source is maintained, and the MMR pathway can start again. This process is described as a futile cycle of repair, and results in the generation of genotoxic DSBs and apoptotic signalling [224,225]. In this way, MMR actually contributes to CDDP cytotoxicity in tumour cells, and its downregulation results in acquired resistance, as defective MMR is associated with decreased CDDP cytotoxicity and poor prognosis in patients [226,227,228].

## 3. Carboplatin, the Second-Generation Pt Agent

**Figure 4:**
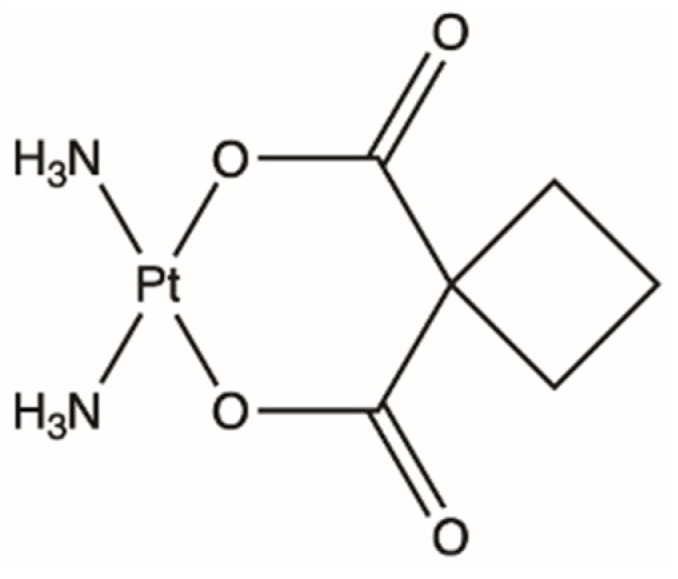
Structural formula of carboplatin. The molecule was adapted from the structure published in the CheBI database [13] using ChemDraw software (v21.0.0.28), PerkinElmer, Waltham, MA, USA.

With the discovery of CDDP and the advantages it gave to the field of chemotherapy and cancer treatment, analogues and other metal compounds were formulated and studied. Moreover, the pressing issue of severe nephrotoxicity caused by CDDP called for a drug that would be as effective as its predecessor but with fewer side effects. Carboplatin (CP), cis-diammine-[1,1-cyclobutanedicarboxylato] platinum(II) (Figure 4), is another globally approved Pt-based chemotherapy agent; CP was synthesized in 1979 and approved for medical use in 1989 [5,6,7,229].

Both CDDP and CP are structurally similar; however, their leaving groups differ. Where CDDP has two chloride ligands, CP has a bidentate cyclobutane dicarboxylate ligand [6]. The difference in the leaving groups explains the difference in toxicity and reactivity of the drugs: the chelate group of CP is more stable than CDDP, thus the aquation step to activate the drug within the cell occurs much slower. Therefore, the reduced reactivity results in less evident ototoxicity and nephrotoxicity [6,230]. CP, like all Pt-agents, has been found to mainly cause damage via forming DNA adducts [6].

### 3.1. Clinical Usage of Carboplatin

CP is used in a wide variety of cancers including ovarian cancer, lung cancer, and certain head-and-neck cancers (Table 1). In particular, it is often used in patients where the side-effects of CDDP are too severe [231]. In ovarian cancer treatment, the combination of CP with paclitaxel has been found to be less toxic than the CDDP-paclitaxel combination and has become the predominant treatment [6]. Combination with docetaxel, cyclophosphamide, as well as use as a single agent have also shown promise in ovarian cancer treatment [232,233]. In many head-and-neck cancers such as retinoblastoma or intracranial germ-cell tumours, CP has been used in combination with etoposide [234]. Additionally, CP has also shown promise in treating a limited number of metastatic breast cancer patients, and is currently being evaluated for the treatment of endometrial cancer [235,236].

### 3.2. The Nuclear Impact of Carboplatin

Once the drug has been activated and enters the nucleus, it can then bind to the DNA and form Pt-DNA adducts [237]. CP can form the same cis-diammine DNA adducts that CDDP does; however, the diverse distribution of adduct types formed and the aforementioned lower reactivity, result in weaker cytotoxicity by CP [238,239,240,241]. While the major adduct of CDDP is the intrastrand 1,2-d(GG) crosslink, CP tends to produce more 1,3-d(GXG) crosslinks [240]. The 1,2-d(GG) crosslink produced by CDDP is considered to be the most cytotoxic, due in part to the severe bending of the DNA helix (55–78°) towards the major groove, and the subsequent unwinding of the helix and opening of the minor groove. Instead, the 1,3-d(GXG) crosslink of CP only bends the helix ~30°, affecting the biological impact of the CP-DNA adduct [240]. Importantly, the bending of the helix allows for HMGB1 protein to stably bind the 1,2-d(GG) Pt-adduct and shield it from DNA repair proteins, unlike with the 1,3-d(GXG) adduct where HMGB1 is unable to bind cleanly; this is likely another reason for the lowered cytotoxicity and reactivity of CP [240,242,243].

**Figure 5:**
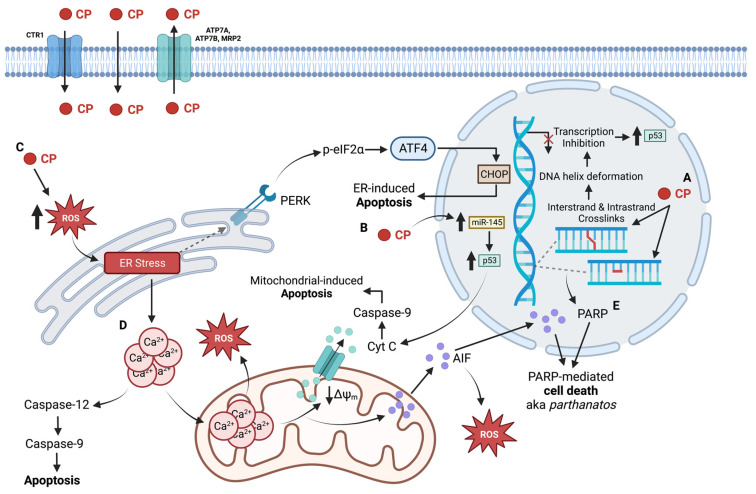
CP targets the nucleus, the mitochondria and ER to induce cell death in its role as an anti-cancer drug. CP enters the cell via passive diffusion and high affinity copper uptake protein 1 (CTR1) and is removed from the cell by efflux transporters, ATP7A, ATP7B, and multidrug resistance protein 2 (MRP2). (**A**) CP forms DNA adducts, intrastrand or interstrand crosslinks, which result in deformation of the DNA helix; transcription inhibition, represented by the red cross, results in replication errors, leading to elevated p53 activity, subsequently activating the apoptosis pathway. (**B**) CP upregulates micro-RNA 145 (miRNA-145) expression, a tumour-suppressor, leading to p53 activation; subsequently p53-mediated mitochondrial-induced apoptosis is triggered. (**C**) CP causes a production of reactive oxygen species (ROS), leading to ER stress, marked by an upregulation of GRP78 and PERK-mediated phosphorylation of eIF2α; ATF4 and CHOP are activated leading to ER-induced apoptosis. (**D**) ER stress also leads to a release of calcium into the cytoplasm, which can directly activate caspase-12 and lead to apoptosis; high levels of calcium are taken up by the mitochondria, depolarizing the membrane. Cytochrome *C* (Cyt *C*) and apoptosis-inducing factor (AIF) are released into the cytoplasm, where Cyt *C* is able to activate caspase-9 and cause mitochondrial-induced apoptosis. AIF also promotes ROS production to further the cycle. (**E**) DNA damage leads to elevated PARP levels, inducing AIF translocation to the nucleus; the combination of both results in PARP-mediated cell death (parthanatos). Created with BioRender.com (Accessed on 11 November, 2022).

Regardless of which adduct CP forms, the resultant crosslinks create deformations in the DNA helix that initiate a similar cell death processes as CDDP; DNA replication and repair can become inhibited leading to transcription errors and inhibition of cell function [6,242] (Figure 5A). As a result of the DNA damage, cell cycle arrest is promoted, proliferation is reduced, and apoptosis is induced [242].

In 2015, Soori et al. demonstrated through spectroscopy that CP had a weaker binding affinity to chromatin-bound DNA compared to free DNA [238]. Furthermore, CP affected both the DNA and the histones of the nucleosomes. The authors noticed that the binding of CP to chromatin does not cause a release of free-DNA or histones, which indicates that the method of binding to chromatin is different from how CP binds to free DNA. They did conclude that CP is likely able to cause its cytotoxic effect partially by forming DNA-histone or histone-histone crosslinks, albeit to a lesser extent than with free-DNA [238].

Once DNA is transcribed into mRNA, it can undergo posttranscriptional regulation by microRNAs (miRNA); short, single-stranded RNA, is endogenous to the cell and non-coding, and acts on the mRNA by targeting the 3′ untranslated region [244]. In particular, miR-145 is a tumour-suppressor miRNA that is often downregulated in many tumours. It is transcriptionally regulated by p53 and inhibit proliferation and migration of the tumour cells [244]. Kilic et al. demonstrated an upregulation of miR-145 in head-and-neck cancer cells that were treated with CP; they also demonstrated a decrease in the expression of the targets of miR-145 (OCT4, SOX2, KLF4, and ABCG2) [244] (Figure 5B). As CP is known to cause DNA lesions, which can trigger p53-signalled cell death, the authors looked at two specific lysine amino acids, K120 and K373, which are acetylated from DNA damage and are known to improve p53 binding to pro-apoptotic genes such as PUMA and Bax [244]. CP was able to increase p53 expression and promote the acetylation of K120 and K373. In order to confirm the association of miR-145 expression with p53 induction, they used a p53 inhibitor, which showed miR-145 suppression as well as increased expression of its target genes [244]. Of the two cell lines they used, HEp-2 cells are wild-type p53 but with a low expression, while FaDu cells have a mutated p53 allele with a functional transcription regulation ability. Interestingly, the mutation in the FaDu cells prevented miR145 expression while the lower p53 expression of HEp2 cells did not have an effect, which indicated that this mechanism is restricted to tumours with functional p53 [244]. Overall, the authors concluded that CP is likely able to induce cell death through elevating miR-145 expression.

### 3.3. Non-Nuclear Targets of Carboplatin

As CDDP is a well-known ER stress inducer, research into assessing the ability of CP to cause ER stress is prudent [5,245]. Brozovic et al. assessed CP treatment in a human laryngeal carcinoma cells parental cell line (HEp2) and a CP-resistant subline (7T) for ER stress, with ROS production as a cause [245]. Rates of CP accumulation, DNA platination, and apoptosis were found to be elevated in the HEp2 parental cell line compared to the 7T subline. Both cell lines showed immediate increases in levels of ER stress markers ATF4 and p-eIF2α when CP entered the cell; however, the levels in the HEp2 cells were significantly higher than in the 7T cells [245]. The downstream ER stress markers, CHOP and GRP78, were found to be elevated in the sensitive parental cells, but not in the resistant 7T cells (Figure 5C). The silencing of CHOP via siRNA to stop ER stress improved survivability of HEp2 cells but not of 7T cells, indicating that CP was directly influencing the ER stress pathway. ROS production appeared earlier in HEp2 cells, and when tempol, an antioxidant, was used as pre-treatment these cells had increased survival unlike the 7T cells which showed no change in survival. The inability of tempol to protect the 7T cells against CP indicates that ROS formation may not be involved in the 7T subline response to the Pt agent [245]. Overall, the results of the study suggest that upon entry into a sensitive cell, CP causes simultaneous DNA damage, ROS production, and activation of the ER stress response [245].

Apoptosis is well-accepted as the most common cell death pathway to occur from a Pt-agent treatment [6,7,246]. Apoptosis can be initiated by the intrinsic or extrinsic pathways; the intrinsic pathway occurs due to oxidative stress or UV radiation which leads to mitochondrial perturbation and eventual caspase-9 activation, while the extrinsic pathway occurs when a death receptor is activated and eventually leads to caspase-8 activation [246,247]. HN-3, a laryngeal carcinoma cell line, was exposed to multiple concentrations of CP and the stages of apoptosis were studied; it was determined that the effects of CP on apoptosis were time- and dose-dependent [246]. Initially, at 12 h post-exposure, the cells began to show increased apoptosis, with elevated intracellular Ca^2+^, increased membrane potential, and mitochondrial depolarization (Figure 5D). From these results, the authors concluded that CP caused the ER to release excess Ca^2+^ which was taken up by the mitochondria and lead to excess ROS production and oxidative stress; treatment with GSH, a ROS scavenger, prevented ER release of Ca^2+^, indicating that oxidative stress was associated with excess Ca^2+^ [246]. Furthermore, as the mitochondria became depolarized, it released cytochrome *C* and apoptosis-inducing factor (AIF) into the cytoplasm, which lead to the activation of caspase-9, triggering apoptosis [246]. AIF was also shown to be involved in a positive feedback loop with the mitochondria to produce increased amounts of ROS to further oxidative stress [246]. At 24 h, 36 h, and 48 h, the cells showed elevated caspase-8 expression, elevated PARP activity, and caspase-9 activation, respectively. Elevated PARP levels resulted in AIF translocating to the nucleus from the mitochondria, which coupled with the CP-induced DNA adducts inhibiting DNA replication and causing replication errors, induces the PARP-mediated cell death pathway coined as “parthanatos” [248,249,250] (Figure 5E). From these results, the authors suggested that the effect of CP requires synergism between caspase-8 and PARP from nuclear damage, and caspase-9 from mitochondrial damage; the role of the ER was also further given importance as it was able to induce apoptosis via caspase-12 activation in the presence of CP [246].

Overall, CP shows similar toxic effects to CDDP, sharing many of the same targets including the DNA, the mitochondria, and the ER. However, the key difference is in the lower cytotoxicity of CP, demonstrated by the lower platination and differing DNA adducts that predominantly forms.

### 3.4. Limitations for the Use of Carboplatin

CP was introduced in the clinic in part to overcome the limitations of CDDP; however, it is not without its own problems. Although CP is able to circumvent the major CDDP side effect of nephrotoxicity due to its lower reactivity, there is still a risk for renal damage to occur [6]. Furthermore, the major dose-limiting side effect of CP is myelosuppression, especially thrombocytopenia over neutropenia or anemia [6,251] (Table 1). Moreover, the structural and functional similarities of CP to CDDP create a high risk for cross-resistance; therefore, patients who have previously been treated with CDDP are unable to be treated with CP [252].

There is also a concern for developing a hypersensitivity reaction to CP treatment, with an estimated incidence rate of up to 35%, depending on the grade and severity [253]. The majority of cases tend to occur in women receiving the drug as a second line treatment, with peak incidence around seven cycles. Both acute and delayed hypersensitivity have been reported, with mild (flushing, rash, nausea, fever, and facial edema) to severe or life-threatening (hypertension/hypotension, tachycardia, wheezing, stridor, laryngeal edema, and shock) symptoms [253].

Compared to the broad spectrum of cancers treated with CDDP, CP has a slightly smaller scope of effectiveness. Both drugs have an intrinsic resistance for colon or rectal cancer, while CP in particular is less effective against testicular germ cell cancer, bladder cancer, and head-and-neck squamous cell carcinoma [6,254].

The mechanisms of resistance that plague CDDP cross over to CP as well, likely due to their similar chemical structure, method of entrance into the cell, activation, and action [6,254].

Influx and efflux of the drug into the cell are impacted due to altered regulation of transporters. While the copper transporters CTR1 and CTR2 preferentially bind CP compared to copper, the efflux transporters ATP7A and ATP7B prefer copper to CP; high levels of the efflux transporters have been associated with poor response to CP in ovarian cancer patients and non-small cell lung cancer [1,255] (Figure 5). A study conducted in A498 renal cancer cells demonstrated that CP has a poorer affinity for CTR1, which gave credence to finding lower intracellular Pt accumulation within the cell; overall DNA platination was also lower after CP treatment [230]. Another aforementioned study on laryngeal carcinoma cells demonstrated similar effects, where CP had a stronger impact in the HEp2 parental cells compared to 7T-resistant cells with respect to CP-induced apoptosis. Total cell and DNA platination was also found to be lower in the 7T subline, leading to fewer intrastrand and interstrand DNA break formation [245]. The higher platination in the HEp2 cell line was supported by higher influx transporter mRNA levels (CTR1 and NHE1 transporters) and lower efflux transporter mRNA levels (ATP7A and MRP2). More CP was retained within the parental cells to cause damage, indicating that a method of resistance to CP occurs by altering levels of influx and efflux transporters [245].

Of the CP-DNA adducts that can form, 90% are intrastrand and are usually repaired by the NER pathway due to their substantial size, while the other 10% are interstrand crosslinks, which are repaired by homologous recombination or the MMR system. Reparation of the DNA adducts, and crosslinks slows down the cell death and can lead to Pt resistance [230,237,256,257]. CP treatment of A498 kidney cancer cells showed an elevation of transcript levels of XPC, DDB1, DDB2, and GADD45A, all of which are indirectly or directly associated with the NER pathway, thus indicating that the NER pathway was activated after CP treatment [230]. Of this pathway, an important protein is cross-complementation group 1 (ERCC1). Ovarian, non-small cell lung, and colorectal cancer patients have demonstrated an inverse relationship between response to CP and ERCC1 mRNA levels [255].

Changes to DNA repair, such as mutations in BRCA or in components of the MMR pathway correlate with CP resistance [1,255]. As DNA mismatch repair is able to identify DNA malformation caused by the CP-DNA adducts, an attempt at repair can lead to signalling for apoptosis; therefore, a loss of MMR lowers the chance of apoptosis to occur [1,255]. Ovarian tumours with a loss of MMR have been associated with resistance to CP treatment [255].

A study looking into a CP-specific prognostic biomarker for resistance in epithelial ovarian cancer suggested that MEK1, involved in the MAPK cascade and the greater EGFR pathway by enhancing proliferation and anti-apoptotic signals, is a strong candidate [1]. Ovarian cancer is predominantly treated with Pt agents, in particular CP. As EGFR is overexpressed in 70% of ovarian cancers and is correlated with chemoresistance and a worse prognosis, the authors looked further into the role of MEK1 [1]. Furthermore, MEK1 activation is thought to cause resistance to Pt-agents due to activation of ERCC1, which has already been correlated with CP resistance [1]. The authors concluded the study by suggesting a concurrent treatment of CP with a MEK1 inhibitor would be beneficial to reduce resistance to the drug [1].

## 4. Oxaliplatin, a Third Attempt

**Figure 6:**
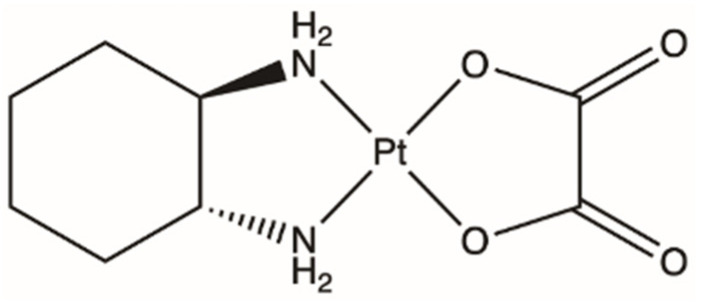
Structural formula of oxaliplatin. The molecule was adapted from the structure published in the CheBI database [13] using ChemDraw software (v21.0.0.28), PerkinElmer, Waltham, MA, USA.

Oxaliplatin (OXP), [oxalate(2-)-O,O’][1R,2R-cyclohexanediamine-N,N’]platinum(II) (Figure 6)*,* was synthesized in 1976 but only approved by the US FDA in 2002 [6,7,258,259]. OXP shows limited or no cross-resistance to CDDP and CP, which makes it a suitable replacement in cancers where an acquired resistance to either drug develops [6,7,260]. In fact, in cancers that CDDP is intrinsically resistant to, such as colorectal cancer, OXP has shown great efficacy [6].

The structure of OXP differs from the other Pt-drugs based on its carrier ligand, 1,2-diaminocyclohexane (DACH) and an oxalate leaving group [6,7]. The DACH ligand is highly lipophilic, which allows the drug to enter the cell very efficiently by passive diffusion and by utilizing non-CTR1 influx transporters [6,7,261]. Hydrolysis of OXP within the cell transforms it into a reactive monoaquated or diaquated species, the only forms able to bind DNA [262,263]. The larger structure of the DACH ligand also allows for OXP to have a different spectrum of action and resistance than CDDP and CP [6,7,261,264,265,266]. Bioactivation of OXP compared to CDDP is slower due to the slow dissociation of the bidentate oxalate leaving group, which results in lower levels of DNA platination and adduct formation [265]. Thus, the oxalate reduces the reactivity of OXP resulting in a significantly lower risk of developing nephrotoxicity or ototoxicity when compared to CDDP [6].

### 4.1. Clinical Usage of Oxaliplatin

OXP was specifically developed to overcome the resistance to CDDP or CP, and in the NCI 60-cell human tumour panel, OXP showed a different pattern of sensitivity than CDDP, indicating a differing array of cancers OXP could treat. Reports have also shown that OXP has promise in cancers for which CDDP is weak against [6,7]. Furthermore, in both in vitro and in vivo models, OXP is shown to be only partially cross-resistant to CP or CDDP, and synergistic with both compounds [260]. In patients who develop hypersensitivity to CDDP and CP, OXP has been studied as a potential substitute as the traditional method of switching CP out for CDDP can result in adverse reactions. The substitution of CDDP or CP with OXP is well tolerated, no additional hypersensitivity occurs, and the general toxicity response is manageable or negligible [260].

OXP has been explicitly approved for the treatment of adjunctive and/or metastatic colorectal cancer by the US FDA and other drug administration bodies globally [6,7,267,268] (Table 1). OXP can be given alone, or provided as part of the FOLFOX combination (5-fluorouracil, leucovorin and OXP) [6,7,267,268]. FOLFOX has been very successful when treating colorectal cancer and is the usual standard of treatment [7,267,268]. Combination of OXP with irinotecan (IROX) or gemcitabine (GEMOX) also shows promise in treatment [7,268,269]. Other combinations that have been studied include OXP with CDDP, paclitaxel, cyclophosphamide, docetaxel, bevacizumab, or pegylated liposomal doxorubicin [260].

Beyond colorectal cancer, there are off-label uses of OXP in the treatment of other digestive system cancers such as esophageal and gastric cancers. Advanced biliary adenocarcinoma, chronic lymphocytic leukemia, advanced pancreatic cancer, and advanced ovarian cancer all list OXP as an indication [6,260,267,268]. Research is ongoing into use in breast cancer and non-small cell lung cancer [6].

### 4.2. Causation of DNA Damage by Oxaliplatin

**Figure 7:**
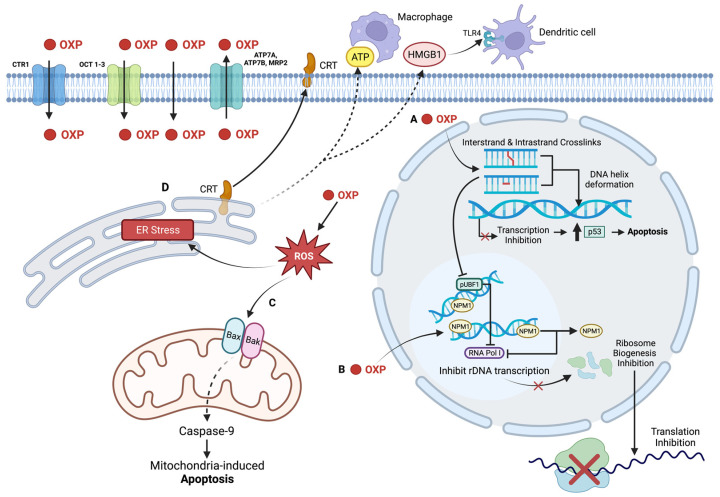
OXP targets the nucleus in two ways, the mitochondria and ER to induce cell death in its role as an anti-cancer drug. OXP enters the cell via passive diffusion and High affinity copper uptake protein 1 (CTR1) and is removed from the cell by efflux transporters, ATP7A, ATP7B, and multidrug resistance protein 2 (MRP2). (**A**) OXP forms DNA adducts, intrastrand or interstrand crosslinks, which result in deformation of the DNA helix; transcription inhibition (represented by a red cross) results in transcription and replication errors, leading to elevated p53 activity, subsequently activating the apoptosis pathway. (**B**) OXP induces nucleophosmin (NPM1) translocation from the nucleolus to the nucleoplasm; this process causes nucleolar segregation, inhibition of RNA Polymerase I (RNA Pol I) activity and formation of fibrillarin caps. RNA Pol I is also inhibited by DNA damage blocking the phosphorylated S484 isoform of UBF1. Subsequently, ribosomal DNA (rDNA) transcription is inhibited (represented by a red cross), and ribosome biogenesis is inhibited. Translation is thus inhibited. (**C**) OXP induces reactive oxygen species (ROS) production leading to the activation of pro-apoptotic factors, Bax and Bak, in a nuclear-independent fashion that leads to caspase-9 activation and subsequent mitochondrial-induced apoptosis. (**D**) ROS production by OXP causes sufficient ER stress to begin apoptosis; during early-apoptosis calreticulin (CRT) and ERp57 translocate to the plasma membrane and act as “find-me” and “eat-me” signals to nearby immune cells; during early-to-late apoptosis and late-apoptosis, ATP and high-grade mobility box 1 (HMGB1), respectively, are secreted into the extracellular environment. ATP acts as another “find-me” signal to monocytes, macrophages and other immune cells, while HMGB1 can bind to TLR4 on dendritic cells to instigate a full immune response. Created with BioRender.com (11 November, 2022).

OXP, like other Pt-based agents, is a DNA-damage inducer via the formation of Pt-DNA adducts, causing growth arrest and triggering apoptosis [230,270] (Figure 7A). OXP has a tendency to form hydrogen bonds on the alternate side of the DNA strand compared to CDDP and CP [72]. This difference allows for a particular interaction between the amine group of the DACH ligand and a 3′ guanine base. The result of that crosslink causes a structural distortion specific to the biologically active form of OXP [72]. In general, DNA-adduct conformations differ between CDDP and OXP due to their ligands; the bulky size of the DACH compared to the two CDDP-chlorides alter the formation of adducts and the response by the cell [72,264].

The adducts formed by OXP, predominantly 1,3-d(GpNpG) and Pt–dG or Pt-dA monoadducts [72,271], often result in a bend to the DNA helix that has a narrow major groove and a wider minor groove which prevents HMGB1 proteins from binding with the OXP-adducts as effectively as with CDDP or even CP [243,265]. Even the 1,2-d(GpG) intrastrand crosslinks that OXP does form are structurally dissimilar to the highly cytotoxic version caused by CDDP, having a smaller dihedral angle, smaller minor groove, and a less bent crystal structure [265].

OXP can enter the cell via passive diffusion and is only known to use the main Pt-influx transporter, CTR1, at lower concentrations, whereas higher concentrations of OXP tends to enter the cell independently of CTR1 [7,230,261,272,273] (Table 2). In fact, upregulation of CTR1 is not correlated with an increase in OXP resistance, indicating a lack of connection between the drug and the receptor [261]. Instead, solute carrier transporters, specifically the organic ion transporter subgroup organic cation transporters 1–3 (OCT 1–3), are more commonly considered the main OXP-influx transporters (Figure 7); albeit the particular affinity of the OXP substrate for each of these transporters within a particular cancer has varied in different reports [261,270,274,275].

The chemical structure of OXP also hinders its ability to form DNA adducts. While the DACH ligand allows for a strong deformation of the DNA strand, the slow dissociation of the oxalate ligand results in lower rates of Pt accumulation. An OXP analogue, [Pt(DACH)Cl_2_], where the oxalate was replaced with chloride leaving groups, like that of CDDP, showed higher rates of adduct formation at equimolar concentrations of OXP [265]. Due to the lower amount of DNA platination, fewer DNA adducts tend to form with OXP treatment. However, OXP is able to more effectively inhibit cell growth compared to CDDP per the same number of DNA adducts [230,261,265,276,277]. Furthermore, OXP is able to form DNA adducts in nearly identical regions to CDDP and maintains a comparable cytotoxicity to CDDP [230,261,264,276,277,278,279].

As mentioned with respect to CDDP and CP, OXP interacts predominantly with chromatin rather than free DNA [238]. Soori et al. showed that OXP has a stronger binding affinity to chromatin compared to CP [238,280]. OXP was also able to maintain a cooperative binding pattern to chromatin, wherein an OXP molecule is more likely to bind to chromatin that already has another OXP bound. This was because OXP had more binding sites for chromatin than CP [238]. Thus, the authors concluded that OXP is able to bind to chromatin better and stronger than CP [238]. OXP is also able to bind to other nucleophilic molecules such as RNA and to a lesser extent, proteins [261,265].

In addition to the inhibition of DNA replication, OXP was shown to also be effective in inhibiting transcription as demonstrated by a study which utilized immobilized DNA templates to create site specific 1,2- and 1,3-crosslinks for CDDP and OXP [72]. Yuan et al. integrated the crosslinks into DNA strands that were encouraged to undergo transcription, both promoter-dependent and independent, in a representative reconstituted system. Of the combinations of crosslinks and drug, 1,3-OXP adducts caused the strongest transcription inhibition comparatively [72].

Bruno et al. assessed RNA interference (RNAi) signatures, comparing CDDP, CP, and OXP, to predict the mechanism of their cytotoxic drug action [281]. The authors compared levels of specific RNAs associated with cell death signalling pathways to create the signatures [281]. In doing so, CDDP and CP were predominantly found to elevate levels of RNA that are associated with DNA crosslink formation. Contrastingly, OXP demonstrated increased RNA involved in transcription and translation inhibition [281].

### 4.3. Effect of Oxaliplatin on Nucleolus Integrity and Ribosome Biogenesis

Ribosome biogenesis is often elevated in cancer and inhibition of the process is currently being studied as a treatment mechanism [281,282]. The steps of ribosome biogenesis include ribosomal RNA (rRNA) transcription, rRNA processing, and ribosomal subunit production [282].

Studies looking at OXP-treated lymphoma and lung adenocarcinoma cells showed levels of pre-RNA that fluctuate in a similar pattern to that of a known ribosome biogenesis stressor, actinomycin D [281,282]. In a similar manner, inhibiting ribosome biogenesis by knocking out RPL11, a critical protein involved in the process, resulted in reduced total p53 and PARP levels in OXP treated cells while CDDP did not show any difference [281]. OXP was able to disrupt protein synthesis affecting the translation machinery in as early as 9 h, whereas CDDP was not. Interestingly, utilization of a translation inhibitor and ribosome biogenesis stressor in combination with OXP showed an antagonistic effect, suggesting that active translation is required for OXP to display its cytotoxic effect [281]. To determine the ability of OXP to induce ribosome biogenesis stress, the gene expression of the translation machinery was assessed. In multiple cancers, gene expression of the machinery was elevated after OXP exposure. The study also went on to suggest that the cells compensate for the stress by increasing the expression of translation molecules implying a “translation addiction” whereby even in the presence of depleted ribosome activity, the cell attempts to instigate translation [281]. The impact of ribosome biogenesis also gives some idea for why OXP is effective in colorectal cancer and less so in ovarian cancer—genes associated with this process were found more prevalently in colorectal cancer tissue samples compared to ovarian cancer [281].

**Figure 8:**
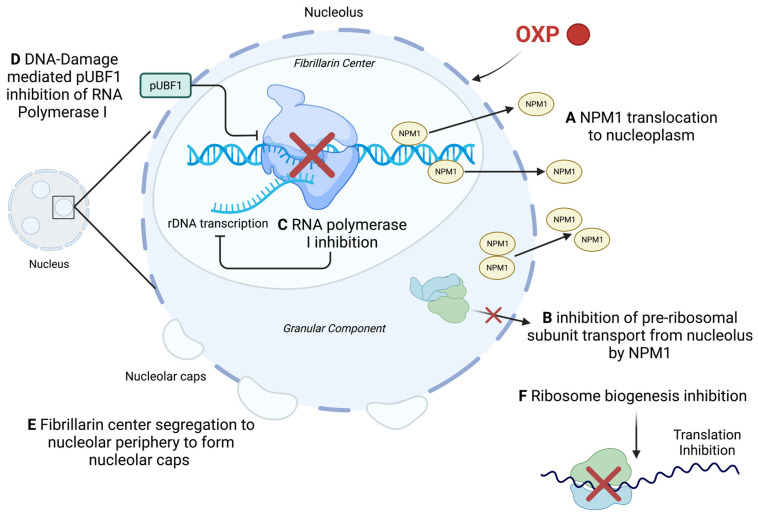
OXP targets the nucleolus and induces nucleolar stress and segregation, and inhibition of ribosome biogenesis. (**A**) OXP induces the translocation of nucleophosmin (NPM1) from the nucleolus to nucleoplasm. (**B**) Pre-ribosomal subunit transport to the nucleoplasm is inhibited by the lack of NPM1. (**C**) Inhibition of rDNA transcription (represented by a red cross) by RNA polymerase I (Pol I) occurs after NPM1 translocation (**D**) OXP-induced DNA crosslinks phosphorylate upstream binding factor 1 (UBF1), preventing RNA polymerase I-mediated rDNA transcription. (**E**) Separation of the fibrillar center and granular component results in segregation of the fibrillarin center along the periphery of the nucleolus, forming nucleolar caps. (**F**) All the effects of OXP in the nucleolus prevent ribosome biogenesis, and subsequently translation inhibition (represented by a red cross). Created with BioRender.com (Accessed on 11 November, 2022).

As ribosome biogenesis occurs within the nucleolus, nucleolar morphology can be assessed for tumour severity, and it is therefore of interest with OXP treatment [282,283]. Ribosome biogenesis stress-inducing agents are known to cause nucleolar segregation, which is characterized by rRNA damage and transcription impairment, separation of the fibrillarin center and granular component, nucleophosmin (NPM1) migration, and nucleolar cap formation [282,283,284] (Figure 8). Studies have indicated that OXP, but not CDDP, is able to induce NPM1 migration from the nucleolus to the nucleoplasm [282,284] (Figure 8A). In particular, the DACH ligand is associated with elevated NPM1 translocation compared to the chloride groups of CDDP [284]. As A549 cell lung carcinoma cells are well-characterized for the nucleolar stress pathway, they were exposed to OXP and CDDP in order to determine how NPM1 migration correlates with ribosome biogenesis [282]. Inhibition of rRNA transcription by RNA polymerase I (Pol I) occurred post-OXP treatment early on compared to equivalent concentrations of CDDP (Figure 8C). This inhibition was found to be correlated with NPM1 translocation prior to the formation of fibrillarin-containing nucleolar caps, which indicated impact on transcription and early processing [282].

Pol I inhibition can occur from ribosomal DNA damage; however, direct DNA damage can also show an increase in Pol I inhibition as with CDDP-treated cells. On the other hand, clinically relevant doses of OXP also increased Pol I inhibition, but DNA damage was not found abundantly surrounding the nucleoli, suggesting that the mechanism of Pol I inhibition was separate from DNA damage and likely correlated with the nucleolar perturbation (NPM1 redistribution) [282] (Figure 8C). The results of the study support the idea that OXP is likely impacting ribosome biogenesis and nucleolar integrity more than causing direct DNA damage [282].

Beyond the nucleosome, there is also the potential for OXP affecting ribosome biogenesis by forming canonical DNA crosslinks such as CDDP and CP [285]. For instance, HCT116 colorectal cancer cells were treated with CDDP and OXP and nuclear proteins were extracted. Specific DNA probes for each drug with 1,2-crosslinks and 1,3-crosslinks were created to bind and isolate the proteins most impacted by each drug-crosslink pair. It was found that across both drugs, the probes for 1,2-crosslinks bound more phosphorylated protein than the 1,3-crosslinks, which coincides with previous reports of 1,2-crosslinks being repaired less effectively and being the dominant lesion of CDDP [230,285]. With respect to CDDP, both 1,2- and 1,3-probes largely isolated mRNA processing clusters of proteins; on the other hand, OXP 1,2-crosslink probes focused on chromatin remodelling proteins while 1,3-crosslinks were split between rRNA processing and mRNA processing [285]. These differences based on drug were also shown in the distinct patterns of protein phosphorylation that were generated. UBF1, a DNA binding protein involved in ribosome biogenesis, was chosen for its functional relevance. Both CDDP and OXP lowered the expression of UBF1 and its S484 phosphorylated isoform; however, OXP managed to do so in a stronger manner than CDDP. These results support the role of OXP in isolating rRNA processing proteins and the elevated cytotoxicity that OXP was shown to have in HCT116 cells compared to CDDP [285]. The S484 phosphorylated isoform of UBF1 is required to interact with RNA Pol I as well as to activate rDNA transcription which has been demonstrated to be downregulated by OXP [285] (Figure 8D). Overall, the study demonstrated the ability of OXP to hinder ribosome biogenesis via DNA adduct formation as well as the superior effect of the drug compared to CDDP in this process [285].

### 4.4. Effect of Oxaliplatin on the Mitochondria 

Although OXP is able to induce cell death through DNA damage, it also plays a larger role outside of DNA. For instance, Gourdier et al. demonstrated that OXP was able to induce mitochondrial apoptosis via activation of pro-apoptotic factors Bax and/or Bak in a p53-independent manner in HCT116 colon carcinoma OXP-sensitive cells; a defect in the Bcl-2 family of proteins was shown to be a critical mechanism for OXP-resistance [261,279,286] (Figure 7C). Additionally, enucleation of the sensitive cells maintained OXP-induced mitochondrial apoptosis and cell death, indicating that the nucleus is not the sole source of OXP activity [279].

### 4.5. Oxaliplatin and Immunogenic Cell Death

A unique mechanism of OXP in treating cancerous cells is the induction of immunogenic cell death (ICD) [268,287]. ICD is the process by which the tumour microenvironment is altered to provide an anti-tumour immunity via a dying tumour cell acting as an “eat-me” signal to nearby immune cells [288]. Previous studies have demonstrated an association between a functional immune system and a successful chemotherapy effect, thus agents able to prepare or bolster the immune system are attractive for cancer research [287]. Well-known ICD inducers include the anthracycline class of drugs, which are able to induce production of activated tumour-specific cytotoxic T-cells in a dendritic cell-dependent manner that can then create an anti-cancer vaccine environment. For ICD, the exposure or release of specific damage-associated molecular patterns (DAMPs) are necessary, in particular: calreticulin (CRT), adenosine triphosphate (ATP), and high-mobility group box 1 (HMGB1) [287,288] (Figure 7D).

ER stress, from drug treatment, results in caspase-8 activation that prompts the translocation of CRT and ERp57 from the ER to the plasma membrane surface, where CRT acts as an “eat-me” or engulfment signal to the nearby immune cells. The translocation of CRT occurs early on in apoptosis, prior to any morphological changes or phosphatidylserine exposure [287,288,289]. Whereas blocking or depletion of CRT is associated with no immunogenicity [290], additional recombinant CRT has been shown to induce ICD in cells that when treated by a particular drug would normally undergo non-immunogenic cell death [287,289,291]. For example, the results of multiple colorectal cancer cell lines treated with CDDP indicate that CDDP cannot translocate sufficient levels of CRT to the plasma membrane surface to induce ICD, however, providing additional CRT generates immunogenicity [287]. ATP and HMGB1 release occur sequentially by the cell later on during apoptosis. ATP acts as a “find-me” signal to monocytes, macrophages and dendritic cells (DC), while HMGB1 causes a strong proinflammatory effect and is able to interact with receptors on DCs such as toll-like receptor 4 (TLR4) [287,288,292]. A loss of TLR4 or a mutated allele, such as those found in some breast cancer patients, diminishes the interaction between OXP and HMGB1; relapse after chemotherapy in these patients is more common than in those with a normal TLR4 allele [287,290,292,293]. Once activated by HMGB1, mature DCs are able to recognize apoptotic cells and initiate the subsequent immune response [288,294]. HMGB1 release is also suggested to instigate general and mitochondrial-specific autophagy of the tumour cell [288].

Colorectal cancer is heavily influenced by the immune system, with the type, density, and location of lymphoid infiltrates present in the tumour bed affecting the development and progression of the disease; thus, cytotoxic T cells and T-helper cells can be beneficial in treating this cancer [287]. As OXP is a potent drug used in treating colorectal cancer, there was reason to believe that OXP is able to induce ICD, and research has supported this conclusion: OXP is able to prepare T cells for interferon-γ production and mediate the anti-cancer vaccine environment in a way that CDDP is unable to [287]. Furthermore, the difference between the toxicities of OXP and CDDP in colorectal cancer cells was found to be due to CRT-exposure; as stated above, CDDP is unable to translocate significant levels of CRT to the plasma membrane while OXP is able to do so. Moreover, removing or inhibiting CRT translocation in OXP-treated cells removed the immunogenicity of the cells [287].

An important point to acknowledge is the requirement of a functioning immune response for OXP efficacy. Progression-free and overall survival is lowered in colorectal cancer patients receiving OXP treatment in those who have a loss-of-function TLR4 mutation, indicating that even with a release of HMGB1 and translocation of CRT, without an active immune system an anti-cancer vaccine environment cannot be formed [287].

In a study looking at colorectal cancer cell lines (HCT116 and HT29CC) and hepatocellular carcinoma (HCC) cell lines (HepG2, SK-Hep1, SNU423, and Hep3b), OXP was shown to induce necrosis and a stronger inflammatory state in the HCC cells compared to the colorectal cell lines which more often underwent apoptosis [294]. In this study, OXP was shown to elevate ROS, which can trigger oxidative stress damage and induce an immune response; however, in the colorectal cell lines the activation of p53 at the correct stage pushed the cell death towards apoptosis. On the other hand, in HCC cells, the p53 activation was not sufficient to counteract the building of ROS levels, thus necrosis and a subsequent inflammatory state were induced [294].

In a 2020 study, murine HCC (H22 and Hepa-16) and human HCC (HepG2 and SMMC7721) cells were exposed to multiple doses of CDDP and OXP each; a single maximally effective dose of 20 µM was chosen to avoid higher dose-side effects [288]. Similar to the preceding colorectal cancer study, CDDP was unable to induce ICD whereas OXP was. Cells with CRT exposed on the plasma membrane, as well as extracellular release of ATP and HMGB1, were observed after OXP treatment. Moreover, signals of immune activation were also elevated: higher numbers of activated mature DCs, CD8^+^ T-cells and reduced Foxp3^+^ T-reg cells, which are a strong indication of survival [288]. The presence of increased CRT exposure as well as mature DCs indicate that OXP is able to induce HCC engulfment by the DCs and create an anti-tumour environment [288]. The study also suggested a method to bolster a cancer-weakened immune system by combining OXP treatment with an anti-PD-1 antibody, which would act to inhibit PD-L1 expression on the tumour cells, thus allowing the immune system to react favourably to OXP-induced ICD. Finally, the researchers shifted from in vitro assays to in vivo syngeneic mouse models, utilizing their tested cell lines to create tumours, which when treated with OXP demonstrated ICD induction [288]. These results, both in vitro and in vivo, support the conclusion that OXP is able to act as an ICD inducer in HCC and colorectal cancer.

### 4.6. Limitations of the Use of Oxaliplatin

As with CDDP and CP, OXP has limitations to its use, either because of side effects, hypersensitivity, or chemoresistance. The major dose-limiting side effect of OXP is peripheral neurotoxicity, occurring acutely or chronically [260,268,295,296,297] (Table 1). Acute OXP-induced peripheral neuropathy (OIPN) occurs in the majority of patients and can be exacerbated by cold stimulation; the symptoms begin to present within hours of the infusion and can last up to 7 days [260,297]. A suggested mechanism of OXP to induce the acute neuropathy is via metabolites of the oxalate group blocking the dorsal root ganglion voltage-gated sodium channel activation and impairing nerve excitability [297]. Acute OIPN occurrence can help to predict the degree of chronic OIPN, which occurs after cumulative dosages of OXP greater than 750–850 mg/m^2^. A suggested mechanism for chronic OIPN is through mitochondrial damage and death of sensory neurons (nerve cell necrosis) [297]. Cases of chronic OIPN tend to be irreversible in less than 5% of cases, and generally within 6–12 months of discontinuation of treatment, the symptoms begin to dissipate [260,268]. Symptoms of OIPN include cold-sensitive peripheral paresthesia, prolonged muscular contractions and fasciculations, and problems with proprioception and sensation loss. Additionally, approximately 60% of patients with chronic OIPN experience “coasting” or prolonged neuropathic symptoms that impair their daily quality of life beyond completion of the last course of chemotherapy [260,297]. While there are no unique risk factors to developing OIPN, patients tend to be of older age, have previous comorbidities, are on opioids or β-blockers, have a high BMI, low serum albumin, low creatinine clearance, and smoke [297]. Furthermore, those who have a baseline neuropathic condition or have specific genetic polymorphisms such as to glutathione transferases, cytochrome p450 enzymes, or OCT2, are known to be more susceptible to developing OIPN. While there is no current treatment, duloxetine is sometimes given to high risk-factor patients treated with OXP in order to avoid developing OIPN [297].

Other major side effects from OXP treatment include gastrointestinal reactions, myelosuppression, and hypersensitivity reactions [260,268,295,296,297] (Table 1). Less major, albeit still common side effects include fatigue and cytopenia [295].

Gastrointestinal problems tend to be of minor concern, limited to mild-to-moderate nausea, vomiting and diarrhea; generally, there are no impacts on the liver or kidney unless previously impaired [260,268]. However, a growing number of colorectal cancer patients present with hepatic sinusoidal obstruction syndrome (HSOS) after being treated with OXP [296]. HSOS is a hepatic vascular disease in which the hepatic sinus endothelial cells are damaged and shed off, blocking the hepatic sinus outflow tract. This obstruction results in liver congestion, reduced liver functional reserve, portal hypertension and worsened postoperative course of patients after liver resection. Clinically, the disease also presents with splenomegaly and thrombocytopenia. Patients diagnosed with OXP-induced HSOS tend to have higher rates of bleeding risk, transfusion rate, reduced tumour response to drug-treatment, longer hospital stays and increased overall morbidity [296]. A correlation between patients treated with OXP and developing HSOS has been demonstrated, with more cases of HSOS being found in patients treated with OXP than those treated with another form of chemotherapy. Incidence and severity of the disease progression is associated with cumulative OXP dose and length of treatment period [296].

With respect to myelosuppression and myelotoxicity, symptoms tend to be moderate: a greater amount of grade 3–4 neutropenia compared to anemia or thrombocytopenia; fever only tends to present in less than 4% of patients [260,268] (Table 1).

Although OXP was designed to overcome hypersensitivity induced by CP or CDDP, patients can still develop hypersensitivity to OXP as the cumulative dosage increases [295]. Notably, across multiple studies the incidence rate of OXP-induced hypersensitivity varies from 2% to 25%, irrespective of the cancer in question. Regardless, less than 1% of cases tend to be life threatening. Most patients present with cutaneous symptoms such as palmar and facial flushing, shortness of breath, nausea, vomiting, and diarrhea; a more severe reaction includes cardiac arrest, dyspnea, myalgia, rhinorrhea, and severe anaphylaxis [295]. The number of chemotherapy sessions a patient has underwent is a significant predictor for risk of developing OXP-induced hypersensitivity. Between 7 and 9 sessions of OXP treatment tends to be the point at which hypersensitivity develops [295]. Developing grade 3 or 4 hypersensitivity is more common in females and in patients with high neutrophils and low monocytes [295].

As with all drugs, resistance can be innately present or acquired over time and use. The mechanisms that increase resistance to OXP include cellular influx/efflux and detoxification, DNA adduct repair, and cell death mechanisms [261,268,270].

Even though OXP is not known to be solely reliant on CTR1 for its predominant influx into cells, OXP is still reliant on transporters. The levels and function of transporters such as soluble carrier transporter family (OCT 1–3) are critical for OXP influx into the cell (Figure 7). Therefore, in models of acquired OXP-resistance, colorectal cancer and ovarian cancer cell lines were induced to have OXP-resistance. Pt-accumulation, DNA adduct formation, transporter expression and DNA repair genes were assessed [270]. Noordhuis et al. concluded that levels of OCT 1–3 gene expression were significantly correlated to Pt-accumulation and the loss or reduction of expression contributed to OXP resistance seen in the induced-resistant cell [270]. Similarly, efflux transporters, such as p-type ATPases (ATP7A and ATP7B), have also shown a potential role in OXP resistance [261,270]. ATP7A, in particular, has been shown to be significantly correlated with the ability of OXP to induce DNA adduct formation. However, that study concluded that the ability of a cell to produce DNA adducts was separate from the cytotoxicity of the drug and suggested that the mechanisms of resistance and action of OXP lay elsewhere [270]. In one clinical trial looking at ATP7A and ATP7B expression levels in colorectal cancer patients treated with first line OXP, patients with lower levels of ATP7B demonstrated better overall survival [261,298]. Another efflux transporter family are the ABC transporters, in particular the ABCC subfamily including MRPs, which have been demonstrated to be associated with OXP resistance [261] (Figure 7). In an ovarian carcinoma in vitro model, MRP1 and MRP4 were found to have higher expression and an altered N-linked glycosylation that resulted in less accumulation of OXP within the cell and increased resistance to the drug [261,299]. In another study, reduction of Na^+^K^+^-ATPase β1 subunit was found to be correlated with OXP resistance in an Na^+^K^+^-ATPase-pump independent manner; this result suggested that exogenous introduction of the β1 subunit was able to increase uptake and sensitization of the cell to OXP, whereas resistance was maintained with a loss of this subunit [261,300].

Another method of resistance to OXP is detoxification, namely by the glutathione system, mediated by the ABCC transporter subfamily [261]. The aquated isomer of OXP is inactivated by glutathione, catalyzed by the glutathione S-transferase enzyme [266]. In vitro results have shown contradictory evidence between levels of glutathione and OXP resistance; for instance, in a study conducted in vitro in six colorectal cancer cell lines that assessed glutathione and glutathione S-transferase levels and activity after OXP and CDDP activity, no correlation could be found between either drug or protein with respect to cytotoxicity or resistance [266]. However, in vivo, some correlation was found in colorectal cancer and gastric cancer patients given OXP. Those with polymorphisms to the GSTP1, a member of the glutathione S-transferases superfamily, causing a reduction in the enzyme, tended to have better overall survival after OXP treatment than those with normal levels of GSTP1 [266]. An important aspect to be noted is the role of the microenvironment with respect to glutathione and OXP resistance. Zhang et al. showed an increase in intracellular glutathione after OXP treatment in chronic lymphocytic leukemia cells; however, this was determined to be due to increased cysteine release into the microenvironment by bone marrow stromal cells, which is taken up by the cancer cells and used to promote glutathione synthesis, bolstering OXP resistance [261,301].

With respect to DNA repair, OXP is unhindered by a deficiency in MMR due to the bulky DACH ligand. OXP-treated cells are therefore able to undergo apoptosis irrespective of MMR pathway functionality [6,7,264,266]. In fact, colorectal cancer, which OXP is used to treat predominantly, is often MMR deficient and this might be a reason why CDDP and CP are unable to treat it effectively [261,302]. Instead, OXP-induced DNA damage is often repaired by the NER system in vitro, in which ERCC1 and XPA have been identified as key mediators [265,266]. In a six-line colorectal cancer in vitro study, OXP cytotoxicity was found to be predicted by ERCC1 levels, and in cells that were able to maintain lower levels of XPA, OXP DNA adducts had a stronger cytotoxic effect [266]. However, ERCC1 and XPA have also been associated with OXP resistance. In vitro *models* of preclinical intrinsically low levels of ERCC1 have been correlated with OXP sensitivity, while elevated levels have been associated with resistance [261,268,303,304,305]. siRNA-mediated silencing of ERCC2 and XPA results in less DNA repair, thus increasing OXP sensitivity and further supporting the association between NER activity and OXP function [261,306]. In patients with stage 3 colorectal cancer treated with OXP, those with ERCC1 positive tumours were found to have a lower 5-year disease free and overall survival than those with ERCC1 negative tumours [307]. OXP is also able to induce free radicals, which create single strand DNA (ssDNA) breaks, resulting in oxidative DNA damage. The base excision repair (BER) system is able to repair these ssDNA breaks, thus an alteration in BER functionality would affect sensitivity to OXP [263]. In colorectal and gastric cell lines, an elevation in polymerase β or polymerase η, major BER DNA polymerases, has been correlated with OXP resistance [261,308,309].

Once OXP has caused its damage and the repair mechanisms have failed to protect the cell, the cell inevitably must undergo cell death; however, there are resistance mechanisms that at this stage too, can interfere with OXP. Alterations to levels of certain pro-apoptotic and anti-apoptotic factors, such as the Bcl-2 family, Bax, inhibitors of apoptosis proteins (IAP) including survivin and BIRC, and p53 have all been correlated with resistance to the intrinsic apoptotic pathway [261,279,286,310,311]. Impairment of the extrinsic apoptotic pathway has also been demonstrated as an OXP resistance mechanism; overexpression of MMP7, which is able to degrade FasL, prevents the progression of the extrinsic apoptosis pathway while silencing of MMP7 restores OXP sensitivity by increasing levels of the Fas receptor bolstering apoptosis [312].

## 5. Overall Conclusions

The introduction of CDDP, CP, and OXP into cancer chemotherapeutics has resulted in dramatic improvements in survival and quality of life for cancer patients. However, in the literature, their nuclear effects have been the predominant focus, while their non-nuclear targets have often been neglected in assessing cytotoxicity. More research needs to be conducted on their cytoplasmic mechanisms of action, as these effects can provide information on the cellular responses and toxicity that may be missed by solely evaluating nuclear damage. Furthermore, elucidating additional cellular targets of these drugs can provide insight into potential methods of reducing side effects and resistance, while enhancing cytotoxicity. A conclusion to be taken from this review is the need for further research to differentiate the actions of CP from those of CDDP. CP is often considered to be equivalent to CDDP and conclusions reached from research on CDDP cytotoxicity is often applied to CP. However, as demonstrated in this review, these drugs do not show identical cytotoxicity towards cancer cells, and while certain mechanisms are common between the two, current research inadequately covers the scope of effects induced by CP. In addition, OXP requires further comparison within CDDP-effective cancers to determine whether it could be used as a low-side effect substitution. Overall, further research needs to be conducted on CP and OXP so as to provide a more well-rounded conclusion of their mechanisms of action, resistance, and what cancers they could be used to treat.

## Figures and Tables

**Table 1:** Comparison of indications and major side effects of cisplatin, carboplatin, and oxaliplatin.

Platinating Agent	Indication	Major Side Effect
Cisplatin	Breast, ovarian, testicular, head-and-neck, esophageal, lung, bladder, brain tumours	Nephrotoxicity, ototoxicity, neurotoxicity, hepatotoxicity, cardiotoxicity
Carboplatin	Ovarian, lung, certain head-and-neck	Myelosuppression (thrombocytopenia)
Oxaliplatin	Colorectal	Peripheral neurotoxicity, Hepatic Sinusoidal obstruction syndrome, myelosuppression

**Table 2:** Comparison of influx, efflux, and cellular targets of cisplatin, carboplatin, and oxaliplatin.

Platinating Agent	Influx	Efflux	Organelle Targets
Cisplatin	CTR1, passive diffusion	ATP7A, ATP7B, MRP2	Nucleus, ER, Mitochondria, Cytoskeleton, Plasma Membrane
Carboplatin	CTR1, passive diffusion		Nucleus, ER, Mitochondria
Oxaliplatin	OCT 1–3, CTR1 (minor), passive diffusion		Nucleus, Nucleolus, ER, Mitochondria

## Data Availability

Not applicable.

## Abbreviations

AIF	Apoptosis-inducing factor
aSMase	Acid sphingomyelinase
ATF6	Activating transcription factor 6
ATP	Adenosine triphosphate
BER	Base excision repair
CDDP	Cisplatin
CHOP	C/EMP homologous protein
CP	Carboplatin
CRT	Calreticulin
CTR1	High affinity copper uptake protein 1
Cyt *C*	Cytochrome *C*
DAMP	Damage-associated molecular patterns
DC	Dendritic cell
DISC	Death-inducing signalling complex
DSB	Double strand breaks
ER	Endoplasmic reticulum
ERCC1	Cross-complementation group 1
FasR	Fas receptor
FDA	US Food and Drug Administration
FOLFOX	5-fluorouracil, leucovorin, oxaliplatin drug combination
GEMOX	Gemcitabine, oxaliplatin drug combination
GSH	Glutathione
HCC	Hepatocellular carcinoma
HMGB1	High-mobility group box 1
HR	Homologous recombination
HSOS	Hepatic sinusoidal obstruction syndrome
IAP	Inhibitors of apoptosis proteins
ICD	Immunogenic cell death
ICL	Interstrand crosslink
IRE1α	Inositol-requiring protein 1α
IROX	Irinotecan, oxaliplatin drug combination
miRNA	microRNA
MMR	Mismatch repair
MRP	Multidrug resistance proteins
mtDNA	Mitochondrial DNA
nDNA	Nuclear DNA
NPM1	Nucleophosmin
Nrf2	NF-E2-related factor 2
OCT	Organic cation transporter
OIPN	Oxaliplatin-induced peripheral neuropathy
OXP	Oxaliplatin
pEIF2α	Phosphorylated eukaryotic translation factor 2α
PERK	Protein kinase RNA-like endoplasmic reticulum kinase
Pol I	RNA Polymerase I
Pt	Platinum
Pt	Platinum
ROS	Reactive oxygen species
rRNA	Ribosomal RNA
ssDNA	Single strand DNA breaks
SSRP1	structure specific recognition protein 1
TFIIH	Transcription factor II H
TLR4	Toll-like receptor 4
UBF	Upstream binding factor
XPC	Xeroderma pigmentosum

## References

[1] Pénzváltó Z., Lánczky A., Lénárt J., Meggyesházi N., Krenács T., Szoboszlai N., Denkert C., Pete I., Győrffy B. (2014). MEK1 is associated with carboplatin resistance and is a prognostic biomarker in epithelial ovarian cancer. BMC Cancer.

[2] DeVita V.T., Chu E. (2008). A History of Cancer Chemotherapy. Cancer Res..

[3] Zhang C., Xu C., Gao X., Yao Q. (2022). Platinum-based drugs for cancer therapy and anti-tumor strategies. Theranostics.

[4] Ndagi U., Mhlongo N., Soliman M.E. (2017). Metal complexes in cancer therapy—An update from drug design perspective. Drug. Des. Devel. Ther..

[5] Famurewa A.C., Mukherjee A.G., Wanjari U.R., Sukumar A., Murali R., Renu K., Vellingiri B., Dey A., Gopalakrishnan A.V. (2022). Repurposing FDA-approved drugs against the toxicity of platinum-based anticancer drugs. Life Sci..

[6] Dilruba S., Kalayda G.V. (2016). Platinum-based drugs: Past, present and future. Cancer Chemotherapy and Pharmacology.

[7] Kelland L. (2007). The resurgence of platinum-based cancer chemotherapy. Nat. Rev. Cancer.

[8] Rottenberg S., Disler C., Perego P. (2020). The rediscovery of platinum-based cancer therapy. Nat. Rev. Cancer.

[9] Dasari S., Tchounwou P.B. (2014). Cisplatin in cancer therapy: Molecular mechanisms of action. Eur. J. Pharmacol..

[10] Karbownik A., Szałek E., Urjasz H., Głęboka A., Mierzwa E., Grzeskowiak E. (2012). The physical and chemical stability of cisplatin (Teva) in concentrate and diluted in sodium chloride 0.9%. Contemp. Oncol..

[11] Zhang J., Ye Z.-W., Tew K.D., Townsend D.M. (2021). Cisplatin chemotherapy and renal function. Adv. Cancer Res..

[12] Hayati F., Hossainzadeh M., Shayanpour S., Abedi-Gheshlaghi Z., Mousavi S.S.B. (2015). Prevention of cisplatin nephrotoxicity. J. Nephropharmacol..

[13] Hastings J., Owen G., Dekker A., Ennis M., Kale N., Muthukrishnan V., Turner S., Swainston N., Mendes P., Steinbeck C. (2015). ChEBI in 2016: Improved services and an expanding collection of metabolites. Nucleic Acids Res..

[14] Peyrone M. (1844). Ueber die Einwirkung des Ammoniaks auf Platinchlorür. Eur. J. Org. Chem..

[15] Rosenberg B., VAN Camp L., Krigas T. (1965). Inhibition of Cell Division in Escherichia coli by Electrolysis Products from a Platinum Electrode. Nature.

[16] Rosenberg B., Vancamp L., Trosko J.E., Mansour V.H. (1969). Platinum Compounds: A New Class of Potent Antitumour Agents. Nature.

[17] Barry N.P.E., Sadler P.J. (2014). 100 years of metal coordination chemistry: From Alfred Werner to anticancer metallodrugs. Pure Appl. Chem..

[18] Higby D.J., Wallace H.J., Albert D.J., Holland J.F. (1974). Diaminodichloroplatinum: A phase I study showing responses in testicular and other tumors. Cancer.

[19] Higby D.J., Wallace H.J., Albert D., Holland J.F. (1974). Diamminodichloroplatinum in the Chemotherapy of Testicular Tumors. J. Urol..

[20] Cersosimo R.J. (1989). Cisplatin neurotoxicity. Cancer Treat. Rev..

[21] Wiltshaw E. (1979). Cisplatin in the treatment of cancer. Platinum Met. Rev..

[22] Daugaard G., Abildgaard U. (1989). Cisplatin nephrotoxicity. Cancer Chemother. Pharmacol..

[23] Jamieson E.R., Lippard S.J. (1999). Structure, Recognition, and Processing of Cisplatin−DNA Adducts. Chem. Rev..

[24] Einhorn L.H. (1990). Treatment of testicular cancer: A new and improved model. J. Clin. Oncol..

[25] Fung C., Dinh P.C., Fossa S.D., Travis L.B. (2019). Testicular Cancer Survivorship. J. Natl. Compr. Cancer Netw..

[26] Boulikas T., Vougiouka M. (2004). Recent clinical trials using cisplatin, carboplatin and their combination chemotherapy drugs (review). Oncol. Rep..

[27] DeConti R.C., Toftness B.R., Lange R.C., A Creasey W. (1973). Clinical and pharmacological studies with cis-diamminedichloroplatinum (II). Cancer Res..

[28] Gale G.R., Morris C.R., Atkins L.M., Smith A.B. (1973). Binding of an antitumor platinum compound to cells as influenced by physical factors and pharmacologically active agents. Cancer Res..

[29] Eljack N.D., Ma H.-Y.M., Drucker J., Shen C., Hambley T.W., New E.J., Friedrich T., Clarke R.J. (2014). Mechanisms of cell uptake and toxicity of the anticancer drug cisplatin. Metallomics.

[30] Binks S.P., Dobrota M. (1990). Kinetics and mechanism of uptake of platinum-based pharmaceuticals by the rat small intestine. Biochem. Pharmacol..

[31] Andrews P.A., Mann S.C., Velury S., Howell S.B., Nicolini M. (1988). Cisplatin Uptake Mediated Cisplatin-Resistance in Human Ovarian Carcinoma Cells. Platinum and Other Metal Coordination Compounds in Cancer Chemotherapy: Proceedings of the Fifth International Symposium on Platinum and Other Metal Coordination Compounds in Cancer Chemotherapy Abano, Padua, Italy, 29 June–2 July 1987.

[32] Andrews P.A., Albright K.D., Howell S.B. (1991). Role of Membrane Ion Transport in Cisplatin Accumulation. Platinum and Other Metal Coordination Compounds in Cancer Chemotherapy.

[33] Ivy K.D., Kaplan J.H. (2013). A Re-Evaluation of the Role of hCTR1, the Human High-Affinity Copper Transporter, in Platinum-Drug Entry into Human Cells. Mol. Pharmacol..

[34] Mann S.C., Andrews P.A., Howell S.B. (1990). Short-term cis-diamminedichloroplatinum(II) accumulation in sensitive and resistant human ovarian carcinoma cells. Cancer Chemother Pharmacol..

[35] Ishida S., Lee J., Thiele D.J., Herskowitz I. (2002). Uptake of the anticancer drug cisplatin mediated by the copper transporter Ctr1 in yeast and mammals. Proc. Natl. Acad. Sci. USA.

[36] Katano K., Kondo A., Safaei R., Holzer A., Samimi G., Mishima M., Kuo Y.-M., Rochdi M., Howell S.B. (2002). Acquisition of resistance to cisplatin is accompanied by changes in the cellular pharmacology of copper. Cancer Res..

[37] Holzer A.K., Samimi G., Katano K., Naerdemann W., Lin X., Safaei R., Howell S.B. (2004). The Copper Influx Transporter Human Copper Transport Protein 1 Regulates the Uptake of Cisplatin in Human Ovarian Carcinoma Cells. Mol. Pharmacol..

[38] Holzer A.K., Katano K., Klomp L.W.J., Howell S.B. (2004). Cisplatin Rapidly Down-regulates Its Own Influx Transporter hCTR1 in Cultured Human Ovarian Carcinoma Cells. Clin. Cancer Res..

[39] Petris M.J., Smith K., Lee J., Thiele D.J. (2003). Copper-stimulated Endocytosis and Degradation of the Human Copper Transporter, hCtr1. J. Biol. Chem..

[40] Kalayda G.V., Wagner C.H., Jaehde U. (2012). Relevance of copper transporter 1 for cisplatin resistance in human ovarian carcinoma cells. J. Inorg. Biochem..

[41] Sinani D., Adle D.J., Kim H., Lee J. (2007). Distinct Mechanisms for Ctr1-mediated Copper and Cisplatin Transport. J. Biol. Chem..

[42] Beretta G.L., Gatti L., Tinelli S., Corna E., Colangelo D., Zunino F., Perego P. (2004). Cellular pharmacology of cisplatin in relation to the expression of human copper transporter CTR1 in different pairs of cisplatin-sensitive and -resistant cells. Biochem. Pharmacol..

[43] Hall M.D., Okabe M., Shen D.-W., Liang X.-J., Gottesman M.M. (2008). The Role of Cellular Accumulation in Determining Sensitivity to Platinum-Based Chemotherapy. Annu. Rev. Pharmacol. Toxicol..

[44] Song I.S., Savaraj N., Siddik Z.H., Liu P., Wei Y., Wu C.J., Kuo M.T. (2004). Role of human copper transporter Ctr1 in the transport of platinum-based antitumor agents in cisplatin-sensitive and cisplatin-resistant cells. Mol. Cancer Ther..

[45] A Andrews P., Mann S.C., Huynh H.H., Albright K.D. (1991). Role of the Na+, K(+)-adenosine triphosphatase in the accumulation of cis-diamminedichloroplatinum(II) in human ovarian carcinoma cells. Cancer Res..

[46] Kishimoto S., Kawazoe Y., Ikeno M., Saitoh M., Nakano Y., Nishi Y., Fukushima S., Takeuchi Y. (2006). Role of Na+, K+-ATPase α1 subunit in the intracellular accumulation of cisplatin. Cancer Chemother. Pharmacol..

[47] A Andrews P., Albright K.D. (1992). Mitochondrial defects in cis-diamminedichloroplatinum(II)-resistant human ovarian carcinoma cells. Cancer Res..

[48] Walker W.F., Johnston I.D.A., Walker W.F., Johnston I.D.A. (1971). 2—Water and Electrolyte Metabolism. The Metabolic Basis of Surgical Care.

[49] El-Khateeb M., Appleton T.G., Gahan L.R., Charles B.G., Berners-Price S.J., Bolton A.-M. (1999). Reactions of cisplatin hydrolytes with methionine, cysteine, and plasma ultrafiltrate studied by a combination of HPLC and NMR techniques. J. Inorg. Biochem..

[50] Pinto A.L., Lippard S.J. (1985). Binding of the antitumor drug cis-diamminedichloroplatinum(II) (cisplatin) to DNA. Biochim. Biophys. Acta.

[51] Miller S.E., House D.A. (1991). The hydrolysis products of cis-diamminedichloroplatinum(II) 5. The anation kinetics of cis-Pt(X)(NH3)2(OH2)+ (X-Cl, OH) with glycine, monohydrogen malonate and chloride. Inorganica Chim. Acta.

[52] Kelland L.R. (1993). New platinum antitumor complexes. Critical Rev. Oncol. /Hematol..

[53] Eastman A. (1983). Characterization of the adducts produced in DNA by cis-diamminedichloroplatinum(II) and cis-dichloro(ethylenediamine)platinum(II). Biochemistry.

[54] Plooy A.C., Fichtinger-Schepman A.M.J., Schutte H.H., Van Dijk M., Lohman P.H. (1985). The quantitative detection of various Pt-DNA-adducts in Chinese hamster ovary cells treated with cisplatin: Application of immunochemical techniques. Carcinogenesis.

[55] Eastman A. (1987). The formation, isolation and characterization of DNA adducts produced by anticancer platinum complexes. Pharmacol. Ther..

[56] Giraud-Panis M.-J., Malinge J.-M., Sedletska Y. (2005). Cisplatin Is a DNA-Damaging Antitumour Compound Triggering Multifactorial Biochemical Responses in Cancer Cells: Importance of Apoptotic Pathways. Curr. Med. Chem. Agents.

[57] Beck D.J., Brubaker R.R. (1973). Effect of cis-platinum(II)diamminodichloride on wild type and deoxyribonucleic acid repair deficient mutants of Escherichia coli. J. Bacteriol..

[58] Fraval H., Rawlings C., Roberts J. (1978). Increased sensitivity of UV-repair-deficient human cells to DNA bound platinum products which unlike thymine dimers are not recognized by an endonuclease extracted from Micrococcus luteus. Mutat. Res. Mol. Mech. Mutagen..

[59] Harder H.C., Smith R.G., Leroy A.F. (1976). Template primer inactivation by cis- and trans-dichlorodiammine platinum for human DNA polymerase alpha, beta, and Rauscher murine leukemia virus reverse transcriptase, as a mechanism of cytotoxicity. Cancer Res..

[60] Pinto A.L., Lippard S.J. (1985). Sequence-dependent termination of in vitro DNA synthesis by cis- and trans-diamminedichloroplatinum (II). Proc. Natl. Acad. Sci. USA.

[61] Sorenson C.M., Eastman A. (1988). Mechanism of cis-diamminedichloroplatinum(II)-induced cytotoxicity: Role of G2 arrest and DNA double-strand breaks. Cancer Res..

[62] Sorenson C.M., Eastman A. (1988). Influence of cis-diamminedichloroplatinum(II) on DNA synthesis and cell cycle progression in excision repair proficient and deficient Chinese hamster ovary cells. Cancer Res..

[63] Sorenson C.M., Barry M.A., Eastman A. (1990). Analysis of Events Associated With Cell Cycle Arrest at G2 Phase and Cell Death Induced by Cisplatin. Gynecol. Oncol..

[64] Jung Y., Lippard S.J. (2003). Multiple States of Stalled T7 RNA Polymerase at DNA Lesions Generated by Platinum Anticancer Agents. J. Biol. Chem..

[65] Tornaletti S., Patrick S.M., Turchi J.J., Hanawalt P.C. (2003). Behavior of T7 RNA Polymerase and Mammalian RNA Polymerase II at Site-specific Cisplatin Adducts in the Template DNA. J. Biol. Chem..

[66] Tremeau-Bravard A., Riedl T., Egly J.-M., Dahmus M.E. (2004). Fate of RNA Polymerase II Stalled at a Cisplatin Lesion. J. Biol. Chem..

[67] Corda Y., Job C., Anin M.F., Leng M., Job D. (1991). Transcription by eucaryotic and procaryotic RNA polymerases of DNA modified at a d(GG) or a d(AG) site by the antitumor drug cis-diamminedichloroplatinum(II). Biochemistry.

[68] Corda Y., Anin M.F., Leng M., Job D. (1992). RNA polymerases react differently at d(ApG) and d(GpG) adducts in DNA modified by cis-diamminedichloroplatinum(II). Biochemistry.

[69] Corda Y., Job C., Anin M.F., Leng M., Job D. (1993). Spectrum of DNA-platinum adduct recognition by prokaryotic and eukaryotic DNA-dependent RNA polymerases. Biochemistry.

[70] Mello J.A., Lippard S.J., Essigmann J.M. (1995). DNA Adducts of cis-Diamminedichloroplatinum(II) and Its Trans Isomer Inhibit RNA Polymerase II Differentially in Vivo. Biochemistry.

[71] Todd R.C., Lippard S.J. (2009). Inhibition of transcription by platinum antitumor compounds. Metallomics.

[72] Vichi P., Coin F., Renaud J., Vermeulen W., Hoeijmakers J., Moras D., Egly J. (1997). Cisplatin- and UV-damaged DNA lure the basal transcription factor TFIID/TBP. EMBO J..

[73] Cullinane C., Mazur S.J., Essigmann J.M., Phillips A.D.R., Bohr V.A. (1999). Inhibition of RNA Polymerase II Transcription in Human Cell Extracts by Cisplatin DNA Damage. Biochemistry.

[74] Treiber D.K., Zhai X., Jantzen H.M., Essigmann J.M. (1994). Cisplatin-DNA adducts are molecular decoys for the ribosomal RNA transcription factor hUBF (human upstream binding factor). Proc. Natl. Acad. Sci. USA.

[75] Zhai X., Beckmann H., Jantzen H.-M., Essigmann J.M. (1998). Cisplatin−DNA Adducts Inhibit Ribosomal RNA Synthesis by Hijacking the Transcription Factor Human Upstream Binding Factor. Biochemistry.

[76] Ise T., Nagatani G., Imamura T., Kato K., Takano H., Nomoto M., Izumi H., Ohmori H., Okamoto T., Ohga T. (1999). Transcription factor Y-box binding protein 1 binds preferentially to cisplatin-modified DNA and interacts with proliferating cell nuclear antigen. Cancer Res..

[77] Yarnell A.T., Oh S., Reinberg D., Lippard S.J. (2001). Interaction of FACT, SSRP1, and the High Mobility Group (HMG) Domain of SSRP1 with DNA Damaged by the Anticancer Drug Cisplatin *. J. Biol. Chem..

[78] Dunham S.U., Lippard S.J. (1997). DNA Sequence Context and Protein Composition Modulate HMG-Domain Protein Recognition of Cisplatin-Modified DNA. Biochemistry.

[79] Mymryk J.S., Zaniewski E., Archer T.K. (1995). Cisplatin Inhibits Chromatin Remodeling, Transcription Factor Binding, and Transcription from the Mouse Mammary Tumor Virus Promoter in vivo. Proc. Natl. Acad. Sci. USA.

[80] E Damsma G., Alt A., Brueckner F., Carell T., Cramer P. (2007). Mechanism of transcriptional stalling at cisplatin-damaged DNA. Nat. Struct. Mol. Biol..

[81] Strauss B.S. (1991). The ‘A rule’ of mutagen specificity: A consequence of DNA polymerase bypass of non-instructional lesions?. BioEssays.

[82] Wu C. (1997). Chromatin Remodeling and the Control of Gene Expression. J. Biol. Chem..

[83] Li B., Carey M., Workman J.L. (2007). The Role of Chromatin during Transcription. Cell.

[84] Ober M., Lippard S.J. (2007). Cisplatin Damage Overrides the Predefined Rotational Setting of Positioned Nucleosomes. J. Am. Chem. Soc..

[85] Ober M., Lippard S.J. (2008). A 1,2-d(GpG) cisplatin intrastrand cross-link influences the rotational and translational setting of DNA in nucleosomes. J. Am. Chem. Soc..

[86] Todd R.C., Lippard S.J. (2010). Consequences of Cisplatin Binding on Nucleosome Structure and Dynamics. Chem. Biol..

[87] Moon H.-M., Park J.-S., Lee I.-B., Kang Y.-I., Jung H.J., An D., Shin Y., Kim M.J., I Kim H., Song J.-J. (2021). Cisplatin fastens chromatin irreversibly even at a high chloride concentration. Nucleic Acids Res..

[88] Charlier C., Kintz P., Dubois N., Plomteux G. (2004). Fatal Overdosage with Cisplatin. J. Anal. Toxicol..

[89] Zhu G., Song L., Lippard S.J. (2013). Visualizing Inhibition of Nucleosome Mobility and Transcription by Cisplatin–DNA Interstrand Crosslinks in Live Mammalian Cells. Cancer Res..

[90] Pucci B., Kasten M., Giordano A. (2000). Cell cycle and apoptosis. Neoplasia.

[91] Donahue B.A., Augot M., Bellon S.F., Treiber D.K., Toney J.H., Lippard S.J., Essigmann J.M. (1990). Characterization of a DNA damage-recognition protein from mammalian cells that binds specifically to intrastrand d(GpG) and d(ApG) DNA adducts of the anticancer drug cisplatin. Biochemistry.

[92] Fink D., Aebi S., Howell S.B. (1998). The role of DNA mismatch repair in drug resistance. Clin. Cancer Res..

[93] Chaney S.G., Vaisman A. (1999). Specificity of platinum–DNA adduct repair. J. Inorg. Biochem..

[94] Appella E., Anderson C.W. (2001). Post-translational modifications and activation of p53 by genotoxic stresses. JBIC J. Biol. Inorg. Chem..

[95] Persons D.L., Yazlovitskaya E.M., Pelling J.C. (2000). Effect of Extracellular Signal-regulated Kinase on p53 Accumulation in Response to Cisplatin. J. Biol. Chem..

[96] Singh M., Chaudhry P., Fabi F., Asselin E. (2013). Cisplatin-induced caspase activation mediates PTEN cleavage in ovarian cancer cells: A potential mechanism of chemoresistance. BMC Cancer.

[97] Cummings B.S., Schnellmann R.G. (2002). Cisplatin-Induced Renal Cell Apoptosis: Caspase 3-Dependent and -Independent Pathways. J. Pharmacol. Exp. Ther..

[98] Jones E.V., Dickman M., Whitmarsh A.J. (2007). Regulation of p73-mediated apoptosis by c-Jun N-terminal kinase. Biochem. J..

[99] Paul I., Chacko A.D., Stasik I., Busacca S., Crawford N., McCoy F., McTavish N., Wilson B., Barr M., O’Byrne K.J. (2012). Acquired differential regulation of caspase-8 in cisplatin-resistant non-small-cell lung cancer. Cell Death Dis..

[100] Mansouri A., Ridgway L.D., Korapati A.L., Zhang Q., Tian L., Wang Y., Siddik Z.H., Mills G.B., Claret F.X. (2003). Sustained Activation of JNK/p38 MAPK Pathways in Response to Cisplatin Leads to Fas Ligand Induction and Cell Death in Ovarian Carcinoma Cells. J. Biol. Chem..

[101] Yang Z., Schumaker L.M., Egorin M.J., Zuhowski E.G., Guo Z., Cullen K.J. (2006). Cisplatin Preferentially Binds Mitochondrial DNA and Voltage-Dependent Anion Channel Protein in the Mitochondrial Membrane of Head and Neck Squamous Cell Carcinoma: Possible Role in Apoptosis. Clin. Cancer Res..

[102] Mandic A., Hansson J., Linder S., Shoshan M.C. (2003). Cisplatin Induces Endoplasmic Reticulum Stress and Nucleus-independent Apoptotic Signaling. J. Biol. Chem..

[103] Yu F., Megyesi J., Price P.M. (2008). Cytoplasmic initiation of cisplatin cytotoxicity. Am. J. Physiol. -Renal Physiol..

[104] Gutekunst M., Oren M., Weilbacher A., Dengler M.A., Markwardt C., Thomale J., Aulitzky W.E., Van Der Kuip H. (2011). p53 Hypersensitivity Is the Predominant Mechanism of the Unique Responsiveness of Testicular Germ Cell Tumor (TGCT) Cells to Cisplatin. PLoS ONE.

[105] Olivero O.A., Chang P.K., Lopez-Larraza D.M., Semino-Mora M.C., Poirier M.C. (1997). Preferential formation and decreased removal of cisplatin–DNA adducts in Chinese hamster ovary cell mitochondrial DNA as compared to nuclear DNA. Mutat. Res. Toxicol. Environ. Mutagen..

[106] Montopoli M., Bellanda M., Lonardoni F., Ragazzi E., Dorigo P., Froldi G., Mammi S., Caparrotta L. (2011). “Metabolic reprogramming” in ovarian cancer cells resistant to cisplatin. Curr. Cancer Drug Targets.

[107] Martins N.M., Santos N.A.G., Curti C., Bianchi M.L.P., Santos A.C. (2007). Cisplatin induces mitochondrial oxidative stress with resultant energetic metabolism impairment, membrane rigidification and apoptosis in rat liver. J. Appl. Toxicol..

[108] Santandreu F.M., Roca P., Oliver J. (2010). Uncoupling protein-2 knockdown mediates the cytotoxic effects of cisplatin. Free Radic. Biol. Med..

[109] Li-ping X., Skrezek C., Wand H., Reibe F. (2000). Mitochondrial dysfunction at the early stage of cisplatin-induced acute renal failure in rats. J. Zhejiang Univ. -Sci..

[110] Marullo R., Werner E., Degtyareva N., Moore B., Altavilla G., Ramalingam S.S., Doetsch P.W. (2013). Cisplatin Induces a Mitochondrial-ROS Response That Contributes to Cytotoxicity Depending on Mitochondrial Redox Status and Bioenergetic Functions. PLoS ONE.

[111] Choi Y.-M., Kim H., Shim W., Anwar M.A., Kwon J.-W., Kwon H.-K., Kim H.J., Jeong H., Kim H.M., Hwang D. (2015). Mechanism of Cisplatin-Induced Cytotoxicity Is Correlated to Impaired Metabolism Due to Mitochondrial ROS Generation. PLoS ONE.

[112] Garrido N., Pérez-Martos A., Faro M., Lou-Bonafonte J.M., Fernández-Silva P., López-Pérez M.J., Montoya J., Enríquez J.A. (2008). Cisplatin-mediated impairment of mitochondrial DNA metabolism inversely correlates with glutathione levels. Biochem. J..

[113] Redza-Dutordoir M., Averill-Bates D.A. (2016). Activation of apoptosis signalling pathways by reactive oxygen species. Biochim. Biophys. Acta (BBA)—Mol. Cell Res..

[114] Fleury C., Mignotte B., Vayssière J.-L. (2002). Mitochondrial reactive oxygen species in cell death signaling. Biochimie.

[115] Wu C.-C., Bratton S.B. (2013). Regulation of the Intrinsic Apoptosis Pathway by Reactive Oxygen Species. Antioxidants Redox Signal..

[116] Halestrap A.P., Woodfield K.-Y., Connern C.P. (1997). Oxidative Stress, Thiol Reagents, and Membrane Potential Modulate the Mitochondrial Permeability Transition by Affecting Nucleotide Binding to the Adenine Nucleotide Translocase *. J. Biol. Chem..

[117] Kagan V.E., Borisenko G.G., Tyurina Y.Y., Tyurin V., Jiang J., Potapovich A.I., Kini V., Amoscato A.A., Fujii Y. (2004). Oxidative lipidomics of apoptosis: Redox catalytic interactions of cytochrome c with cardiolipin and phosphatidylserine. Free. Radic. Biol. Med..

[118] Kleih M., Böpple K., Dong M., Gaißler A., Heine S., Olayioye M.A., Aulitzky W.E., Essmann F. (2019). Direct impact of cisplatin on mitochondria induces ROS production that dictates cell fate of ovarian cancer cells. Cell Death Dis..

[119] Schwarz D.S., Blower M.D. (2016). The endoplasmic reticulum: Structure, function and response to cellular signaling. Cell. Mol. Life Sci..

[120] Avril T., Vauléon E., Chevet E. (2017). Endoplasmic reticulum stress signaling and chemotherapy resistance in solid cancers. Oncogenesis.

[121] Hetz C. (2012). The unfolded protein response: Controlling cell fate decisions under ER stress and beyond. Nat. Rev. Mol. Cell Biol..

[122] Lee A.S. (2005). The ER chaperone and signaling regulator GRP78/BiP as a monitor of endoplasmic reticulum stress. Methods.

[123] Costa-Mattioli M., Walter P. (2020). The integrated stress response: From mechanism to disease. Science.

[124] Tabas I., Ron D. (2011). Integrating the mechanisms of apoptosis induced by endoplasmic reticulum stress. Nat. Cell Biol..

[125] Hu H., Tian M., Ding C., Yu S. (2019). The C/EBP Homologous Protein (CHOP) Transcription Factor Functions in Endoplasmic Reticulum Stress-Induced Apoptosis and Microbial Infection. Front. Immunol..

[126] Yoneda T., Imaizumi K., Oono K., Yui D., Gomi F., Katayama T., Tohyama M. (2001). Activation of Caspase-12, an Endoplastic Reticulum (ER) Resident Caspase, through Tumor Necrosis Factor Receptor-associated Factor 2-dependent Mechanism in Response to the ER Stress *. J. Biol. Chem..

[127] Nakagawa T., Zhu H., Morishima N., Li E., Xu J., Yankner B.A., Yuan J. (2000). Caspase-12 mediates endoplasmic-reticulum-specific apoptosis and cytotoxicity by amyloid-β. Nature.

[128] Yang H., Niemeijer M., van de Water B., Beltman J.B. (2020). ATF6 Is a Critical Determinant of CHOP Dynamics during the Unfolded Protein Response. iScience.

[129] Xu Y., Yu H., Qin H., Kang J., Yu C., Zhong J., Su J., Li H., Sun L. (2012). Inhibition of autophagy enhances cisplatin cytotoxicity through endoplasmic reticulum stress in human cervical cancer cells. Cancer Lett..

[130] Xu Y., Wang C., Su J., Xie Q., Ma L., Zeng L., Yu Y., Liu S., Li S., Li Z. (2015). Tolerance to endoplasmic reticulum stress mediates cisplatin resistance in human ovarian cancer cells by maintaining endoplasmic reticulum and mitochondrial homeostasis. Oncol. Rep..

[131] Liu H., Baliga R. (2005). Endoplasmic Reticulum Stress–Associated Caspase 12 Mediates Cisplatin-Induced LLC-PK1 Cell Apoptosis. J. Am. Soc. Nephrol..

[132] Shen L., Wen N., Xia M., Zhang Y., Liu W., Xu Y., Sun L. (2016). Calcium efflux from the endoplasmic reticulum regulates cisplatin-induced apoptosis in human cervical cancer HeLa cells. Oncol. Lett..

[133] Li C., Wei J., Li Y., He X., Zhou Q., Yan J., Zhang J., Liu Y., Shu H.-B. (2013). Transmembrane Protein 214 (TMEM214) Mediates Endoplasmic Reticulum Stress-induced Caspase 4 Enzyme Activation and Apoptosis. J. Biol. Chem..

[134] Harwood S.M., Yaqoob M.M., A Allen D. (2005). Caspase and calpain function in cell death: Bridging the gap between apoptosis and necrosis. Ann. Clin. Biochem. Int. J. Biochem. Lab. Med..

[135] Danese A., Leo S., Rimessi A., Wieckowski M.R., Fiorica F., Giorgi C., Pinton P. (2021). Cell death as a result of calcium signaling modulation: A cancer-centric prospective. Biochim. et Biophys. Acta (BBA) Mol. Cell Res..

[136] Giorgi C., Marchi S., Pinton P. (2018). The machineries, regulation and cellular functions of mitochondrial calcium. Nat. Rev. Mol. Cell Biol..

[137] Akman M., Belisario D.C., Salaroglio I.C., Kopecka J., Donadelli M., De Smaele E., Riganti C. (2021). Hypoxia, endoplasmic reticulum stress and chemoresistance: Dangerous liaisons. J. Exp. Clin. Cancer Res..

[138] Tian J., Liu R., Qu Q. (2017). Role of endoplasmic reticulum stress on cisplatin resistance in ovarian carcinoma. Oncol. Lett..

[139] Rashid H.-O., Yadav R.K., Kim H.-R., Chae H.-J. (2015). ER stress: Autophagy induction, inhibition and selection. Autophagy.

[140] Burger K.N., Staffhorst R.W., De Kruijff B. (1999). Interaction of the anti-cancer drug cisplatin with phosphatidylserine in intact and semi-intact cells. Biochim. Biophys. Acta (BBA) -Biomembr..

[141] Speelmans G., Sips W.H., Grisel R.J., Staffhorst R.W., Fichtinger-Schepman A.M.J., Reedijk J., de Kruijff B. (1996). The interaction of the anti-cancer drug cisplatin with phospholipids is specific for negatively charged phospholipids and takes place at low chloride ion concentration. Biochim. Biophys. Acta (BBA) -Biomembr..

[142] Suwalsky M., Hernández P., Villena F., Sotomayor C.P. (2000). The Anticancer Drug Cisplatin Interacts with the Human Erythrocyte Membrane. Z. Naturforschung C.

[143] Ramachandran S., Quist A.P., Kumar S., Lal R. (2006). Cisplatin Nanoliposomes for Cancer Therapy:  AFM and Fluorescence Imaging of Cisplatin Encapsulation, Stability, Cellular Uptake, and Toxicity. Langmuir.

[144] Wang K., Lu J., Li R. (1996). The events that occur when cisplatin encounters cells. Coord. Chem. Rev..

[145] Lacour S., Hammann A., Grazide S., Lagadic-Gossmann D., Athias A., Sergent O., Laurent G., Gambert P., Solary E., Dimanche-Boitrel M.-T. (2004). Cisplatin-Induced CD95 Redistribution into Membrane Lipid Rafts of HT29 Human Colon Cancer Cells. Cancer Res..

[146] Rebillard A., Tekpli X., Meurette O., Sergent O., LeMoigne-Muller G., Vernhet L., Gorria M., Chevanne M., Christmann M., Kaina B. (2007). Cisplatin-Induced Apoptosis Involves Membrane Fluidification via Inhibition of NHE1 in Human Colon Cancer Cells. Cancer Res..

[147] Zhang Y., Zeng W., Jia F., Ye J., Zhao Y., Luo Q., Zhu Z., Wang F. (2020). Cisplatin-induced alteration on membrane composition of A549 cells revealed by ToF-SIMS. Surf. Interface Anal..

[148] Nganga R., Oleinik N., Ogretmen B., Chalfant C.E., Fisher P.B. (2018). Chapter One—Mechanisms of Ceramide-Dependent Cancer Cell Death. Advances in Cancer Research.

[149] Fife C.M., A McCarroll J., Kavallaris M. (2014). Movers and shakers: Cell cytoskeleton in cancer metastasis. Br. J. Pharmacol..

[150] Köpf-Maier P., Mühlhausen S. (1992). Changes in the cytoskeleton pattern of tumor cells by cisplatin in vitro. Chem. Interact..

[151] Zeidan Y.H., Jenkins R.W., Hannun Y.A. (2008). Remodeling of cellular cytoskeleton by the acid sphingomyelinase/ceramide pathway. J. Cell Biol..

[152] Min Y.J., Poruchynsky M.S., Sackett D.L., Murphy B., Fojo T. (2008). Cisplatin markedly enhances microtubule depolymerization in A549 cell line compared with oxaliplatin. Cancer Res..

[153] Tulub A.A., Stefanov V.E. (2001). Cisplatin stops tubulin assembly into microtubules. A new insight into the mechanism of antitumor activity of platinum complexes. Int. J. Biol. Macromol..

[154] Raudenska M., Kratochvilova M., Vicar T., Gumulec J., Balvan J., Polanska H., Pribyl J., Masarik M. (2019). Cisplatin enhances cell stiffness and decreases invasiveness rate in prostate cancer cells by actin accumulation. Sci. Rep..

[155] Pierson-Marchandise M., Gras V., Moragny J., Micallef J., Gaboriau L., Picard S., Choukroun G., Masmoudi K., Liabeuf S., The French National Network of Pharmacovigilance Centres (2017). The drugs that mostly frequently induce acute kidney injury: A case − noncase study of a pharmacovigilance database. Br. J. Clin. Pharmacol..

[156] Hoek J., Bloemendal K.M., van der Velden L.A., van Diessen J.N., van Werkhoven E., Klop W.M., Tesselaar M.E. (2016). Nephrotoxicity as a Dose-Limiting Factor in a High-Dose Cisplatin-Based Chemoradiotherapy Regimen for Head and Neck Carcinomas. Cancers.

[157] Hanigan M.H., Devarajan P. (2003). Cisplatin nephrotoxicity: Molecular mechanisms. Cancer Ther..

[158] Gonzalez-Vitale J.C., Hayes D.M., Cvitkovic E., Sternberg S.S. (1977). The renal pathology in clinical trials of Cis-platinum (II) diamminedichloride. Cancer.

[159] Arany I., Safirstein R.L. (2003). Cisplatin nephrotoxicity. Semin. Nephrol..

[160] Megyesi J., Safirstein R.L., Price P.M. (1998). Induction of p21WAF1/CIP1/SDI1 in kidney tubule cells affects the course of cisplatin-induced acute renal failure. J. Clin. Investig..

[161] Tsuruya K., Ninomiya T., Tokumoto M., Hirakawa M., Masutani K., Taniguchi M., Fukuda K., Kanai H., Kishihara K., Hirakata H. (2003). Direct involvement of the receptor-mediated apoptotic pathways in cisplatin-induced renal tubular cell death. Kidney Int..

[162] Baliga R., Ueda N., Walker P.D., Shah S.V. (1999). Oxidant Mechanisms in Toxic Acute Renal Failure*. Drug Metab. Rev..

[163] Ramesh G., Reeves W.B. (2006). Cisplatin Increases TNF-α mRNA Stability in Kidney Proximal Tubule Cells. Ren. Fail..

[164] Ramesh G., Reeves W.B. (2002). TNF-α mediates chemokine and cytokine expression and renal injury in cisplatin nephrotoxicity. J. Clin. Investig..

[165] Ramesh G., Reeves W. (2003). TNFR2-mediated apoptosis and necrosis in cisplatin-induced acute renal failure. Am. J. Physiol. Physiol..

[166] Winston J.A., Safirstein R. (1985). Reduced renal blood flow in early cisplatin-induced acute renal failure in the rat. Am. J. Physiol. Physiol..

[167] Offerman J.J.G., Meijer S., Sleijfer D.T., Mulder N.H., Donker A.J.M., Koops H.S., Van Der Hem G.K. (1984). Acute effects of cis-diamminedichloroplatinum (CDDP) on renal function. Cancer Chemother. Pharmacol..

[168] Callejo A., Sedó-Cabezón L., Juan I.D., Llorens J. (2015). Cisplatin-Induced Ototoxicity: Effects, Mechanisms and Protection Strategies. Toxics.

[169] Knight K.R.G., Kraemer D.F., Neuwelt E.A. (2005). Ototoxicity in Children Receiving Platinum Chemotherapy: Underestimating a Commonly Occurring Toxicity That May Influence Academic and Social Development. J. Clin. Oncol..

[170] Kushner B.H., Budnick A., Kramer K., Modak S., Cheung N.-K.V. (2006). Ototoxicity from high-dose use of platinum compounds in patients with neuroblastoma. Cancer.

[171] Kopke R., Allen K.A., Henderson D., Hoffer M., Frenz D., VAN DE Water T. (1999). A Radical Demise: Toxins and Trauma Share Common Pathways in Hair Cell Death. Ann. N. Y. Acad. Sci..

[172] Breglio A.M., Rusheen A.E., Shide E.D., Fernandez K.A., Spielbauer K.K., McLachlin K.M., Hall M.D., Amable L., Cunningham L.L. (2017). Cisplatin is retained in the cochlea indefinitely following chemotherapy. Nat. Commun..

[173] Filipski K.K., Mathijssen R.H., Mikkelsen T.S., Schinkel A.H., Sparreboom A. (2009). Contribution of Organic Cation Transporter 2 (OCT2) to Cisplatin-Induced Nephrotoxicity. Clin. Pharmacol. Ther..

[174] Ciarimboli G., Deuster D., Knief A., Sperling M., Holtkamp M., Edemir B., Pavenstädt H., Lanvers-Kaminsky C., am Zehnhoff-Dinnesen A., Schinkel A.H. (2010). Organic cation transporter 2 mediates cisplatin-induced oto- and nephrotoxicity and is a target for protective interventions. Am. J. Pathol..

[175] Gregg R.W., Molepo J.M., Monpetit V.J., Mikael N.Z., Redmond D., Gadia M., Stewart D.J. (1992). Cisplatin neurotoxicity: The relationship between dosage, time, and platinum concentration in neurologic tissues, and morphologic evidence of toxicity. J. Clin. Oncol..

[176] Starobova H., Vetter I. (2017). Pathophysiology of Chemotherapy-Induced Peripheral Neuropathy. Front. Mol. Neurosci..

[177] Lomonaco M., Milone M., Batocchi A.P., Padua L., Restuccia D., Tonali P. (1992). Cisplatin neuropathy: Clinical course and neurophysiological findings. J. Neurol..

[178] Siegal T., Haim N. (1990). Cisplatin-induced peripheral neuropathy. Frequent off-therapy deterioration, demyelinating syndromes, and muscle cramps. Cancer.

[179] Kerckhove N., Collin A., Condé S., Chaleteix C., Pezet D., Balayssac D. (2017). Long-Term Effects, Pathophysiological Mechanisms, and Risk Factors of Chemotherapy-Induced Peripheral Neuropathies: A Comprehensive Literature Review. Front. Pharmacol..

[180] Cavalli F., Tschopp L., Sonntag R.W., Zimmermann A. (1978). A case of liver toxicity following cis-dichlorodiammineplatinum(II) treatment. Cancer Treat. Rep..

[181] Hu Y., Sun B., Zhao B., Mei D., Gu Q., Tian Z. (2018). Cisplatin-induced cardiotoxicity with midrange ejection fraction: A case report and review of the literature. Medicine.

[182] Astolfi L., Ghiselli S., Guaran V., Chicca M., Simoni E., Olivetto E., Lelli G., Martini A. (2013). Correlation of adverse effects of cisplatin administration in patients affected by solid tumours: A retrospective evaluation. Oncol. Rep..

[183] Batchelor D. (2001). Hair and cancer chemotherapy: Consequences and nursing care—A literature study. Eur. J. Cancer Care.

[184] Kartalou M., Essigmann J.M. (2001). Mechanisms of resistance to cisplatin. Mutation Res. /Fundam. Molecular Mech. Mutagen..

[185] Kilari D., Guancial E., Kim E.S. (2016). Role of copper transporters in platinum resistance. World J. Clin. Oncol..

[186] Öhrvik H., Logeman B., Turk B., Reinheckel T., Thiele D.J. (2016). Cathepsin Protease Controls Copper and Cisplatin Accumulation via Cleavage of the Ctr1 Metal-binding Ectodomain. J. Biol. Chem..

[187] Inoue Y., Matsumoto H., Yamada S., Kawai K., Suemizu H., Gika M., Takanami I., Iwazaki M., Nakamura M. (2010). Association of ATP7A expression and in vitro sensitivity to cisplatin in non-small cell lung cancer. Oncol. Lett..

[188] Samimi G., Varki N.M., Wilczynski S., Safaei R., Alberts D.S., Howell S.B. (2003). Increase in expression of the copper transporter ATP7A during platinum drug-based treatment is associated with poor survival in ovarian cancer patients. Clin. Cancer Res..

[189] Yang T., Chen M., Chen T., Thakur A. (2015). Expression of the copper transporters hCtr1, ATP7A and ATP7B is associated with the response to chemotherapy and survival time in patients with resected non-small cell lung cancer. Oncol. Lett..

[190] Song L., Li Y., Li W., Wu S., Li Z. (2013). miR-495 Enhances the Sensitivity of Non-Small Cell Lung Cancer Cells to Platinum by Modulation of Copper-Transporting P-type Adenosine Triphosphatase A (ATP7A). J. Cell. Biochem..

[191] Yu Z., Cao W., Ren Y., Zhang Q., Liu J. (2020). ATPase copper transporter A, negatively regulated by miR-148a-3p, contributes to cisplatin resistance in breast cancer cells. Clin. Transl. Med..

[192] Hinoshita E., Uchiumi T., Taguchi K., Kinukawa N., Tsuneyoshi M., Maehara Y., Sugimachi K., Kuwano M. (2000). Increased expression of an ATP-binding cassette superfamily transporter, multidrug resistance protein 2, in human colorectal carcinomas. Clin. Cancer Res..

[193] Yamasaki M., Makino T., Masuzawa T., Kurokawa Y., Miyata H., Takiguchi S., Nakajima K., Fujiwara Y., Matsuura N., Mori M. (2011). Role of multidrug resistance protein 2 (MRP2) in chemoresistance and clinical outcome in oesophageal squamous cell carcinoma. Br. J. Cancer.

[194] Amable L. (2016). Cisplatin resistance and opportunities for precision medicine. Pharmacol. Res..

[195] Rudin C.M., Yang Z., Schumaker L.M., VanderWeele D., Newkirk K., Egorin M.J., Zuhowski E.G., Cullen K.J. (2003). Inhibition of glutathione synthesis reverses Bcl-2-mediated cisplatin resistance. Cancer Res..

[196] Chen H.H.W., Kuo M.T. (2010). Role of Glutathione in the Regulation of Cisplatin Resistance in Cancer Chemotherapy. Met. Based Drugs.

[197] Byun S.-S., Kim S.W., Choi H., Lee C., Lee E. (2005). Augmentation of cisplatin sensitivity in cisplatin-resistant human bladder cancer cells by modulating glutathione concentrations and glutathione-related enzyme activities. BJU Int..

[198] Rocha C.R.R., Garcia C.C.M., Vieira D.B., Quinet A., de Andrade-Lima L.C., Munford V., Belizário J.E., Menck C.F.M. (2014). Glutathione depletion sensitizes cisplatin- and temozolomide-resistant glioma cells in vitro and in vivo. Cell Death Dis..

[199] Hayden A., Douglas J., Sommerlad M., Andrews L., Gould K., Hussain S., Thomas G.J., Packham G., Crabb S.J. (2014). The Nrf2 transcription factor contributes to resistance to cisplatin in bladder cancer. Urol. Oncol. Semin. Orig. Investig..

[200] Li D., Hong X., Zhao F., Ci X., Zhang S. (2021). Targeting Nrf2 may reverse the drug resistance in ovarian cancer. Cancer Cell Int..

[201] Wangy X.-J., Suny Z., Villeneuve N.F., Zhang S., Zhao F., Li Y., Chen W., Yi X., Zheng W., Wondrak G.T. (2008). Nrf2 enhances resistance of cancer cells to chemotherapeutic drugs, the dark side of Nrf2. Carcinog..

[202] Jiang T., Harder B., Rojo de la Vega M., Wong P.K., Chapman E., Zhang D.D. (2015). p62 links autophagy and Nrf2 signaling. Free Radic. Biol. Med..

[203] Yu H., Su J., Xu Y., Kang J., Li H., Zhang L., Yi H., Xiang X., Liu F., Sun L. (2011). p62/SQSTM1 involved in cisplatin resistance in human ovarian cancer cells by clearing ubiquitinated proteins. Eur. J. Cancer.

[204] Bao L.-J., Jaramillo M.C., Zhang Z.-B., Zheng Y.-X., Yao M., Zhang D.D., Yi X.-F. (2014). Nrf2 induces cisplatin resistance through activation of autophagy in ovarian carcinoma. Int. J. Clin. Exp. Pathol..

[205] Rocha C.R.R., Silva M.M., Quinet A., Cabral-Neto J.B., Menck C.F.M. (2018). DNA repair pathways and cisplatin resistance: An intimate relationship. Clinics.

[206] Schärer O.D. (2013). Nucleotide Excision Repair in Eukaryotes. Cold Spring Harb. Perspect. Biol..

[207] Duan M., Ulibarri J., Liu K.J., Mao P. (2020). Role of Nucleotide Excision Repair in Cisplatin Resistance. Int. J. Mol. Sci..

[208] Ng J.M., Vermeulen W., van der Horst G.T., Bergink S., Sugasawa K., Vrieling H., Hoeijmakers J.H. (2003). A novel regulation mechanism of DNA repair by damage-induced and RAD23-dependent stabilization of xeroderma pigmentosum group C protein. Genes Dev..

[209] Nocentini S., Coin F., Saijo M., Tanaka K., Egly J.-M. (1997). DNA Damage Recognition by XPA Protein Promotes Efficient Recruitment of Transcription Factor II H. J. Biol. Chem..

[210] Yokoi M., Masutani C., Maekawa T., Sugasawa K., Ohkuma Y., Hanaoka F. (2000). The Xeroderma Pigmentosum Group C Protein Complex XPC-HR23B Plays an Important Role in the Recruitment of Transcription Factor IIH to Damaged DNA. J. Biol. Chem..

[211] Oksenych V., de Jesus B.B., Zhovmer A., Egly J.-M., Coin F. (2009). Molecular insights into the recruitment of TFIIH to sites of DNA damage. EMBO J..

[212] Coin F., Oksenych V., Egly J.-M. (2007). Distinct Roles for the XPB/p52 and XPD/p44 Subcomplexes of TFIIH in Damaged DNA Opening during Nucleotide Excision Repair. Mol. Cell.

[213] Houtsmuller A.B., Rademakers S., Nigg A.L., Hoogstraten D., Hoeijmakers J.H., Vermeulen W. (1999). Action of DNA repair endonuclease ERCC1/XPF in living cells. Science.

[214] Ito S., Kuraoka I., Chymkowitch P., Compe E., Takedachi A., Ishigami C., Coin F., Egly J.-M., Tanaka K. (2007). XPG Stabilizes TFIIH, Allowing Transactivation of Nuclear Receptors: Implications for Cockayne Syndrome in XP-G/CS Patients. Mol. Cell.

[215] Kemp M.G., Reardon J.T., Lindsey-Boltz L., Sancar A. (2012). Mechanism of Release and Fate of Excised Oligonucleotides during Nucleotide Excision Repair. J. Biol. Chem..

[216] Ogi T., Limsirichaikul S., Overmeer R.M., Volker M., Takenaka K., Cloney R., Nakazawa Y., Niimi A., Miki Y., Jaspers N.G. (2010). Three DNA Polymerases, Recruited by Different Mechanisms, Carry Out NER Repair Synthesis in Human Cells. Mol. Cell.

[217] Ferry K.V., Hamilton T.C., Johnson S.W. (2000). Increased nucleotide excision repair in cisplatin-resistant ovarian cancer cells: Role of ercc1–xpf. Biochem. Pharmacol..

[218] Prakash R., Zhang Y., Feng W., Jasin M. (2015). Homologous Recombination and Human Health: The Roles of BRCA1, BRCA2, and Associated Proteins. Cold Spring Harb. Perspect. Biol..

[219] Toh M., Ngeow J. (2021). Homologous Recombination Deficiency: Cancer Predispositions and Treatment Implications. Oncologist.

[220] Sakai W., Swisher E.M., Karlan B.Y., Agarwal M.K., Higgins J., Friedman C., Villegas E., Jacquemont C., Farrugia D.J., Couch F.J. (2008). Secondary mutations as a mechanism of cisplatin resistance in BRCA2-mutated cancers. Nature.

[221] Swisher E.M., Sakai W., Karlan B.Y., Wurz K., Urban N., Taniguchi T. (2008). Secondary BRCA1 Mutations in BRCA1-Mutated Ovarian Carcinomas with Platinum Resistance. Cancer Res..

[222] Sawant A., Kothandapani A., Zhitkovich A., Sobol R.W., Patrick S.M. (2015). Role of mismatch repair proteins in the processing of cisplatin interstrand cross-links. DNA Repair.

[223] Yamada M., O’Regan E., Brown R., Karran P. (1997). Selective recognition of a cisplatin-DNA adduct by human mismatch repair proteins. Nucleic Acids Res..

[224] Stojic L., Brun R., Jiricny J. (2004). Mismatch repair and DNA damage signalling. DNA Repair.

[225] Topping R.P., Wilkinson J.C., Scarpinato K.D. (2009). Mismatch Repair Protein Deficiency Compromises Cisplatin-induced Apoptotic Signaling. J. Biol. Chem..

[226] Aebi S., Kurdi-Haidar B., Gordon R., Cenni B., Zheng H., Fink D., Christen R.D., Boland C.R., Koi M., Fishel R. (1996). Loss of DNA mismatch repair in acquired resistance to cisplatin. Cancer Res..

[227] Fink D., Zheng H., Nebel S., Norris P.S., Aebi S., Lin T.P., Nehmé A., Christen R.D., Haas M., MacLeod C.L. (1997). In vitro and in vivo resistance to cisplatin in cells that have lost DNA mismatch repair. Cancer Res..

[228] Fink D., Nebel S., Norris P.S., Baergen R.N., Wilczynski S.P., Costa M.J., Haas M., Cannistra S.A., Howell S.B. (1998). Enrichment for DNA mismatch repair-deficient cells during treatment with cisplatin. Int. J. Cancer.

[229] Monneret C. (2011). Platinum anticancer drugs. From serendipity to rational design. Annales Pharmaceutiques Françaises.

[230] Schoch S., Gajewski S., Rothfuß J., Hartwig A., Köberle B. (2020). Comparative Study of the Mode of Action of Clinically Approved Platinum-Based Chemotherapeutics. Int. J. Mol. Sci..

[231] van der Vijgh W.J. (1991). Clinical pharmacokinetics of carboplatin. Clin. Pharmacokinet.

[232] Vasey P.A., Jayson G.C., Gordon A., Gabra H., Coleman R., Atkinson R., Parkin D., Paul J., Hay A., Kaye S.B. (2004). Phase III Randomized Trial of Docetaxel-Carboplatin Versus Paclitaxel-Carboplatin as First-line Chemotherapy for Ovarian Carcinoma. JNCI.

[233] Swenerton K., Jeffrey J., Stuart G., Roy M., Krepart G., Carmichael J., Drouin P., Stanimir R., O’Connell G., MacLean G. (1992). Cisplatin-cyclophosphamide versus carboplatin-cyclophosphamide in advanced ovarian cancer: A randomized phase III study of the National Cancer Institute of Canada Clinical Trials Group. J. Clin. Oncol..

[234] Doz F., Neuenschwander S., Plantaz D., Courbon B., Gentet J.C., Bouffet E., Mosseri V., Vannier J.P., Mechinaud F., Desjardins L. (1995). Etoposide and carboplatin in extraocular retinoblastoma: A study by the Societe Francaise d'Oncologie Pediatrique. J. Clin. Oncol..

[235] Robert N., Leyland-Jones B., Asmar L., Belt R., Ilegbodu D., Loesch D., Raju R., Valentine E., Sayre R., Cobleigh M. (2006). Randomized phase III study of trastuzumab, paclitaxel, and carboplatin compared with trastuzumab and paclitaxel in women with HER-2-overexpressing metastatic breast cancer. J. Clin. Oncol..

[236] Miller D.S., Filiaci V.L., Mannel R.S., Cohn D.E., Matsumoto T., Tewari K.S., DiSilvestro P., Pearl M.L., Argenta P.A., Powell M.A. (2020). Carboplatin and Paclitaxel for Advanced Endometrial Cancer: Final Overall Survival and Adverse Event Analysis of a Phase III Trial (NRG Oncology/GOG0209). J. Clin. Oncol..

[237] Martin L.P., Hamilton T.C., Schilder R.J. (2008). Platinum Resistance: The Role of DNA Repair Pathways. Clin. Cancer Res..

[238] Soori H., Rabbani-Chadegani A., Davoodi J. (2015). Exploring binding affinity of oxaliplatin and carboplatin, to nucleoprotein structure of chromatin: Spectroscopic study and histone proteins as a target. Eur. J. Med. Chem..

[239] Chaney S.G., Campbell S., Temple B., Bassett E., Wu Y., Faldu M. (2004). Protein interactions with platinum–DNA adducts: From structure to function. J. Inorg. Biochem..

[240] Teuben J.-M., Bauer C., Wang A.H.J., Reedijk J. (1999). Solution Structure of a DNA Duplex Containing a *cis*-Diammineplatinum(II) 1,3-d(GTG) Intrastrand Cross-Link, a Major Adduct in Cells Treated with the Anticancer Drug Carboplatin. Biochemistry.

[241] Stefanou D.T., Souliotis V.L., Zakopoulou R., Liontos M., Bamias A. (2021). DNA Damage Repair: Predictor of Platinum Efficacy in Ovarian Cancer?. Biomedicines.

[242] Wang D., Lippard S.J. (2005). Cellular processing of platinum anticancer drugs. Nat. Rev. Drug Discov..

[243] Rabik C.A., Dolan M.E. (2007). Molecular mechanisms of resistance and toxicity associated with platinating agents. Cancer Treat. Rev..

[244] Kilic A., Barlak N., Sanli F., Aytatli A., Capik O., Karatas O.F. (2020). Mode of action of carboplatin via activating p53/miR-145 axis in head and neck cancers. Laryngoscope.

[245] Brozovic A., Vuković L., Polančac D.S., Arany I., Köberle B., Fritz G., Fiket Ž., Majhen D., Ambriović-Ristov A., Osmak M. (2013). Endoplasmic Reticulum Stress Is Involved in the Response of Human Laryngeal Carcinoma Cells to Carboplatin but Is Absent in Carboplatin-Resistant Cells. PLoS ONE.

[246] Shen B., Mao W., Ahn J., Chung P., He P. (2018). Mechanism of HN-3 cell apoptosis induced by carboplatin: Combination of mitochondrial pathway associated with Ca^2+^ and the nucleus pathways. Mol. Med. Rep..

[247] Kashyap D., Garg V.K., Goel N., Donev R. (2021). Chapter Four—Intrinsic and extrinsic pathways of apoptosis: Role in cancer development and prognosis. Advances in Protein Chemistry and Structural Biology.

[248] David K.K., Andrabi S.A., Dawson T.M., Dawson V.L. (2009). Parthanatos, a messenger of death. Front Biosci..

[249] A Fatokun A., Dawson V.L., Dawson T.M. (2014). Parthanatos: Mitochondrial-linked mechanisms and therapeutic opportunities. Br. J. Pharmacol..

[250] Andrabi S.A., Kim N.S., Yu S.-W., Wang H., Koh D.W., Sasaki M., Klaus J.A., Otsuka T., Zhang Z., Koehler R.C. (2006). Poly(ADP-ribose) (PAR) polymer is a death signal. Proc. Natl. Acad. Sci. USA.

[251] Cheng Y.-J., Wu R., Cheng M.-L., Du J., Hu X.-W., Yu L., Zhao X.-K., Yao Y.-M., Long Q.-Z., Zhu L.-L. (2016). Carboplatin-induced hematotoxicity among patients with non-small cell lung cancer: Analysis on clinical adverse events and drug-gene interactions. Oncotarget.

[252] Gore M., Fryatt I., Wiltshaw E., Dawson T., Robinson B., Calvert A. (1989). Cisplatin/carboplatin cross-resistance in ovarian cancer. Br. J. Cancer.

[253] Pandey A., Bhosale B., Pandita V., Singh A., Ghosh J., Ghosh J., Bajpai J. (2014). Carboplatin hypersensitivity in relapsed ovarian carcinoma: A therapeutic challenge. Indian J. Med. Paediatr. Oncol..

[254] Stewart D.J. (2007). Mechanisms of resistance to cisplatin and carboplatin. Crit. Rev. Oncol..

[255] De Sousa G.F., Wlodarczyk S.R., Monteiro G. (2014). Carboplatin: Molecular mechanisms of action associated with chemoresistance. Braz. J. Pharm. Sci..

[256] Gavande N.S., VanderVere-Carozza P.S., Hinshaw H.D., Jalal S.I., Sears C.R., Pawelczak K.S., Turchi J.J. (2016). DNA repair targeted therapy: The past or future of cancer treatment?. Pharmacol. Ther..

[257] Huang Y., Li L. (2013). DNA crosslinking damage and cancer—A tale of friend and foe. Transl. Cancer Res..

[258] Wheate N.J., Walker S., Craig G.E., Oun R. (2010). The status of platinum anticancer drugs in the clinic and in clinical trials. Dalton Trans..

[259] Graham J., Muhsin M., Kirkpatrick P. (2004). Oxaliplatin. Nature Rev. Drug Discov..

[260] Bogliolo S., Cassani C., Gardella B., Musacchi V., Babilonti L., Venturini P.-L., Ferrero S., Spinillo A. (2015). Oxaliplatin for the treatment of ovarian cancer. Expert Opin. Investig. Drugs.

[261] Martinez-Balibrea E., Martínez-Cardús A., Ginés A., de Porras V.R., Moutinho C., Layos L., Manzano J.L., Bugés C., Bystrup S., Esteller M. (2015). Tumor-Related Molecular Mechanisms of Oxaliplatin Resistance. Mol. Cancer Ther..

[262] Zhang H.-Y., Liu Y.-R., Ji C., Li W., Dou S.-X., Xie P., Wang W.-C., Zhang L.-Y., Wang P.-Y. (2013). Oxaliplatin and Its Enantiomer Induce Different Condensation Dynamics of Single DNA Molecules. PLoS ONE.

[263] Malina J., Novakova O., Vojtiskova M., Natile G., Brabec V. (2007). Conformation of DNA GG Intrastrand Cross-Link of Antitumor Oxaliplatin and Its Enantiomeric Analog. Biophys. J..

[264] Zhang C.-M., Lv J.-F., Gong L., Yu L.-Y., Chen X.-P., Zhou H.-H., Fan L. (2016). Role of Deficient Mismatch Repair in the Personalized Management of Colorectal Cancer. Int. J. Environ. Res. Public Heal..

[265] Ahmad S. (2010). Platinum-DNA Interactions and Subsequent Cellular Processes Controlling Sensitivity to Anticancer Platinum Complexes. Chem. Biodivers..

[266] Arnould S., Hennebelle I., Canal P., Bugat R., Guichard S. (2003). Cellular determinants of oxaliplatin sensitivity in colon cancer cell lines. Eur. J. Cancer.

[267] Devanabanda B., Kasi A. (2022). StatPearls.

[268] Alcindor T., Beauger N. (2011). Oxaliplatin: A Review in the Era of Molecularly Targeted Therapy. Curr. Oncol..

[269] Demols A., Peeters M., Polus M., Marechal R., Gay F., Monsaert E., Hendlisz A., Van Laethem J.L. (2006). Gemcitabine and oxaliplatin (GEMOX) in gemcitabine refractory advanced pancreatic adenocarcinoma: A phase II study. Br. J. Cancer.

[270] Noordhuis P., Laan A.C., van de Born K., Honeywell R.J., Peters G.J. (2019). Coexisting Molecular Determinants of Acquired Oxaliplatin Resistance in Human Colorectal and Ovarian Cancer Cell Lines. Int. J. Mol. Sci..

[271] Hah S.S., Sumbad R.A., White R.W.D.V., Turteltaub K.W., Henderson P.T. (2007). Characterization of Oxaliplatin−DNA Adduct Formation in DNA and Differentiation of Cancer Cell Drug Sensitivity at Microdose Concentrations. Chem. Res. Toxicol..

[272] Holzer A.K., Manorek G.H., Howell S.B. (2006). Contribution of the Major Copper Influx Transporter CTR1 to the Cellular Accumulation of Cisplatin, Carboplatin, and Oxaliplatin. Mol. Pharmacol..

[273] Larson C.A., Blair B.G., Safaei R., Howell S.B. (2008). The Role of the Mammalian Copper Transporter 1 in the Cellular Accumulation of Platinum-Based Drugs. Mol. Pharmacol..

[274] Yonezawa A., Masuda S., Yokoo S., Katsura T., Inui K.-I. (2006). Cisplatin and Oxaliplatin, but Not Carboplatin and Nedaplatin, Are Substrates for Human Organic Cation Transporters (SLC22A1–3 and Multidrug and Toxin Extrusion Family). J. Pharmacol. Exp. Ther..

[275] Fujita S., Hirota T., Sakiyama R., Baba M., Ieiri I. (2018). Identification of drug transporters contributing to oxaliplatin-induced peripheral neuropathy. J. Neurochem..

[276] Woynarowski J.M., Faivre S., Herzig M.C., Arnett B., Chapman W.G., Trevino A.V., Raymond E., Chaney S.G., Vaisman A., Varchenko M. (2000). Oxaliplatin-Induced Damage of Cellular DNA. Mol. Pharmacol..

[277] Woynarowski J.M., Chapman W.G., Napier C., Herzig M.C.S., Juniewicz P. (1998). Sequence- and Region-Specificity of Oxaliplatin Adducts in Naked and Cellular DNA. Mol. Pharmacol..

[278] Faivre S., Chan D., Salinas R., Woynarowska B., Woynarowski J.M. (2003). DNA strand breaks and apoptosis induced by oxaliplatin in cancer cells. Biochem. Pharmacol..

[279] Gourdier I., Crabbe L., Andreau K., Pau B., Kroemer G. (2004). Oxaliplatin-induced mitochondrial apoptotic response of colon carcinoma cells does not require nuclear DNA. Oncogene.

[280] Lakowicz J.R., Quenching of Fluorescence (2006). Principles of Fluorescence Spectroscopy.

[281] Bruno P.M., Liu Y., Park G.Y., Murai J., Koch C.E., Eisen T.J., Pritchard J.R., Pommier Y., Lippard S.J., Hemann M.T. (2017). A subset of platinum-containing chemotherapeutic agents kills cells by inducing ribosome biogenesis stress. Nat. Med..

[282] Sutton E.C., DeRose V.J. (2021). Early nucleolar responses differentiate mechanisms of cell death induced by oxaliplatin and cisplatin. J. Biol. Chem..

[283] Yang K., Yang J., Yi J. (2018). Nucleolar Stress: Hallmarks, sensing mechanism and diseases. Cell Stress.

[284] Sutton E.C., McDevitt C.E., Prochnau J.Y., Yglesias M.V., Mroz A.M., Yang M.C., Cunningham R.M., Hendon C.H., DeRose V.J. (2019). Nucleolar Stress Induction by Oxaliplatin and Derivatives. J. Am. Chem. Soc..

[285] Yuan X., Zhang W., He Y., Yuan J., Song D., Chen H., Qin W., Qian X., Yu H., Guo Z. (2020). Proteomic analysis of cisplatin- and oxaliplatin-induced phosphorylation in proteins bound to Pt–DNA adducts. Metallomics.

[286] Gourdier I., Del Rio M., Crabbé L., Candeil L., Copois V., Ychou M., Auffray C., Martineau P., Mechti N., Pommier Y. (2002). Drug specific resistance to oxaliplatin is associated with apoptosis defect in a cellular model of colon carcinoma. FEBS Lett..

[287] Tesniere A., Schlemmer F., Boige V., Kepp O., Martins I., Ghiringhelli F., Aymeric L., Michaud M., Apetoh L., Barault L. (2009). Immunogenic death of colon cancer cells treated with oxaliplatin. Oncogene.

[288] Zhu H., Shan Y., Ge K., Lu J., Kong W., Jia C. (2020). Oxaliplatin induces immunogenic cell death in hepatocellular carcinoma cells and synergizes with immune checkpoint blockade therapy. Cell. Oncol..

[289] Zitvogel L., Kepp O., Senovilla L., Menger L., Chaput N., Kroemer G. (2010). Immunogenic Tumor Cell Death for Optimal Anticancer Therapy: The Calreticulin Exposure Pathway. Clin. Cancer Res..

[290] Panaretakis T., Kepp O., Brockmeier U., Tesniere A., Bjorklund A.-C., Chapman D.C., Durchschlag M., Joza N., Pierron G., van Endert P. (2009). Mechanisms of pre-apoptotic calreticulin exposure in immunogenic cell death. EMBO J..

[291] Obeid M., Tesniere A., Ghiringhelli F., Fimia G.M., Apetoh L., Perfettini J.-L., Castedo M., Mignot G., Panaretakis T., Casares N. (2006). Calreticulin exposure dictates the immunogenicity of cancer cell death. Nat. Med..

[292] Stojanovska V., McQuade R.M., Fraser S., Prakash M., Gondalia S., Stavely R., Palombo E., Apostolopoulos V., Sakkal S., Nurgali K. (2018). Oxaliplatin-induced changes in microbiota, TLR4+ cells and enhanced HMGB1 expression in the murine colon. PLoS ONE.

[293] Apetoh L., Ghiringhelli F., Tesniere A., Obeid M., Ortiz C., Criollo A., Mignot G., Maiuri M.C., Ullrich E., Saulnier P. (2007). Toll-like receptor 4–dependent contribution of the immune system to anticancer chemotherapy and radiotherapy. Nat. Med..

[294] Lim S.-C., Choi J.E., Kang H.S., SI H. (2010). Ursodeoxycholic acid switches oxaliplatin-induced necrosis to apoptosis by inhibiting reactive oxygen species production and activating p53-caspase 8 pathway in HepG2 hepatocellular carcinoma. Int. J. Cancer.

[295] Rogers B.B., Cuddahy T., Briscella C., Ross N., Olszanski A., Denlinger C.S. (2019). Oxaliplatin: Detection and Management of Hypersensitivity Reactions. Clin. J. Oncol. Nurs..

[296] Zhu C., Ren X., Liu D., Zhang C. (2021). Oxaliplatin-induced hepatic sinusoidal obstruction syndrome. Toxicology.

[297] Yang Y., Zhao B., Gao X., Sun J., Ye J., Li J., Cao P. (2021). Targeting strategies for oxaliplatin-induced peripheral neuropathy: Clinical syndrome, molecular basis, and drug development. J. Exp. Clin. Cancer Res..

[298] Martinez-Balibrea E., Martínez-Cardús A., Musulen E., Ginés A., Manzano J.L., Aranda E., Plasencia C., Neamati N., Abad A. (2009). Increased levels of copper efflux transporter ATP7B are associated with poor outcome in colorectal cancer patients receiving oxaliplatin-based chemotherapy. Int. J. Cancer.

[299] Beretta G.L., Benedetti V., Cossa G., Assaraf Y.G., Bram E., Gatti L., Corna E., Carenini N., Colangelo D., Howell S.B. (2010). Increased levels and defective glycosylation of MRPs in ovarian carcinoma cells resistant to oxaliplatin. Biochem. Pharmacol..

[300] Tummala R., Wolle D., Barwe S.P., Sampson V.B., Rajasekaran A.K., Pendyala L. (2009). Expression of Na,K-ATPase-beta(1) subunit increases uptake and sensitizes carcinoma cells to oxaliplatin. Cancer Chemother. Pharmacol..

[301] Zhang W., Trachootham D., Liu J., Chen G., Pelicano H., Garcia-Prieto C., Lu W., Burger J.A., Croce C.M., Plunkett W. (2012). Stromal control of cystine metabolism promotes cancer cell survival in chronic lymphocytic leukaemia. Nature.

[302] Cassidy J. (2000). Review of oxaliplatin: An active platinum agent in colorectal cancer. Int. J. Clin. Pract..

[303] Boyer J., McLean E.G., Aroori S., Wilson P., McCulla A., Carey P.D., Longley D.B., Johnston P.G. (2004). Characterization of p53 Wild-Type and Null Isogenic Colorectal Cancer Cell Lines Resistant to 5-Fluorouracil, Oxaliplatin, and Irinotecan. Clin. Cancer Res..

[304] Hatch S.B., Swift L.P., Caporali S., Carter R., Hill E.J., MacGregor T.P., D’Atri S., Middleton M.R., McHugh P.J., Sharma R.A. (2013). XPF protein levels determine sensitivity of malignant melanoma cells to oxaliplatin chemotherapy: Suitability as a biomarker for patient selection. Int. J. Cancer.

[305] Bohanes P., LaBonte M.J., Lenz H.-J. (2011). A Review of Excision Repair Cross-complementation Group 1 in Colorectal Cancer. Clin. Color. Cancer.

[306] Graf N., Ang W.H., Zhu G., Myint M., Lippard S.J. (2011). Role of Endonucleases XPF and XPG in Nucleotide Excision Repair of Platinated DNA and Cisplatin/Oxaliplatin Cytotoxicity. ChemBioChem.

[307] Li P., Fang Y.J., Li F., Ou Q.J., Chen G., Ma G. (2013). ERCC1, defective mismatch repair status as predictive biomarkers of survival for stage III colon cancer patients receiving oxaliplatin-based adjuvant chemotherapy. Br. J. Cancer.

[308] Yang J., Parsons J., Nicolay N.H., Caporali S., Harrington C.F., Singh R., Finch D., D’Atri S., Farmer P.B., Johnston P.G. (2009). Cells deficient in the base excision repair protein, DNA polymerase beta, are hypersensitive to oxaliplatin chemotherapy. Oncogene.

[309] Teng K.-Y., Qiu M.-Z., Li Z.-H., Luo H.-Y., Zeng Z.-L., Luo R.-Z., Zhang H.-Z., Wang Z.-Q., Li Y.-H., Xu R.-H. (2010). DNA polymeraseη protein expression predicts treatment response and survival of metastatic gastric adenocarcinoma patients treated with oxaliplatin-based chemotherapy. J. Transl. Med..

[310] Wen K., Fu Z., Wu X., Feng J., Chen W., Qian J. (2013). Oct-4 is required for an antiapoptotic behavior of chemoresistant colorectal cancer cells enriched for cancer stem cells: Effects associated with STAT3/Survivin. Cancer Lett..

[311] van Houdt W.J., Emmink B.L., Pham T.V., Piersma S.R., Verheem A., Vries R.G., Fratantoni S.A., Pronk A., Clevers H., Rinkes I.B. (2011). Comparative Proteomics of Colon Cancer Stem Cells and Differentiated Tumor Cells Identifies BIRC6 as a Potential Therapeutic Target. Mol. Cell. Proteom..

[312] Almendro V., Ametller E., García-Recio S., Collazo O., Casas I., Augé J.M., Maurel J., Gascón P. (2009). The Role of MMP7 and Its Cross-Talk with the FAS/FASL System during the Acquisition of Chemoresistance to Oxaliplatin. PLoS ONE.

